# A PDGFRα-driven mouse model of glioblastoma reveals a stathmin1-mediated mechanism of sensitivity to vinblastine

**DOI:** 10.1038/s41467-018-05036-4

**Published:** 2018-08-06

**Authors:** Hyun Jung Jun, Vicky A. Appleman, Hua-Jun Wu, Christopher M. Rose, Javier J. Pineda, Alan T. Yeo, Bethany Delcuze, Charlotte Lee, Aron Gyuris, Haihao Zhu, Steve Woolfenden, Agnieszka Bronisz, Ichiro Nakano, Ennio A. Chiocca, Roderick T. Bronson, Keith L. Ligon, Jann N. Sarkaria, Steve P. Gygi, Franziska Michor, Timothy J. Mitchison, Al Charest

**Affiliations:** 1 Cancer Research Institute, Beth Israel Deaconess Medical Center, Boston, MA 02215 USA; 2 Department of Biostatistics and Computational Biology, Dana-Farber Cancer Institute, Boston, MA 02215 USA; 3 Department of Biostatistics, Harvard T. H. Chan School of Public Health, Boston, MA 02115 USA; 4 Department of Cell Biology, Harvard Medical School, Boston, MA 02215 USA; 5 Department of Systems Biology, Harvard Medical School, Boston, MA 02215 USA; 6 Sackler School of Graduate Studies, Tufts University School of Medicine, Boston, MA 02111 USA; 7 Molecular Oncology Research Institute, Tufts Medical Center, Boston, MA 02111 USA; 8 Harvey Cushing Neuro-Oncology Laboratories, Department of Neurosurgery, Brigham and Women’s Hospital, Harvard Medical School, Boston, MA 02215 USA; 9 Department of Neurosurgery and Comprehensive Cancer Center, University of Alabama at Birmingham, Birmingham, AL 35243 USA; 10 Rodent Histopathology Core, Dana-Farber/Harvard Cancer Center, Boston, MA 02215 USA; 11 Department of Oncologic Pathology, Dana-Farber Cancer Institute, Boston, MA 02215 USA; 12 Department of Radiation Oncology, Mayo Clinic, Rochester, MN 55902 USA; 13 Department of Medicine, Harvard Medical School, Boston, MA 02215 USA

## Abstract

Glioblastoma multiforme (GBM) is an aggressive primary brain cancer that includes focal amplification of PDGFRα and for which there are no effective therapies. Herein, we report the development of a genetically engineered mouse model of GBM based on autocrine, chronic stimulation of overexpressed PDGFRα, and the analysis of GBM signaling pathways using proteomics. We discover the tubulin-binding protein Stathmin1 (STMN1) as a PDGFRα phospho-regulated target, and that this mis-regulation confers sensitivity to vinblastine (VB) cytotoxicity. Treatment of PDGFRα-positive mouse and a patient-derived xenograft (PDX) GBMs with VB in mice prolongs survival and is dependent on STMN1. Our work reveals a previously unconsidered link between PDGFRα activity and STMN1, and highlight an STMN1-dependent cytotoxic effect of VB in GBM.

## Abstract (web summary)

Amplification of PDGFRα is a common alteration in glioblastoma. In this study, the authors develop a genetically engineered mouse model of GBM based on autocrine, chronic stimulation of overexpressed PDGFR and discover Stathmin1 as an important PDGFRα regulated-protein involved in the response to vinstabline.

## Introduction

Extensive molecular characterization of glioblastoma multiforme (GBM) achieved through The Cancer Genome Atlas (TCGA)^1–3^ has revealed a number of genes that are significantly mutated, namely *PTEN, TP53, EGFR, PIK3CA, PIK3R1, NF1, RB1*, and *PDGFRA*, where *EGFR* and *PDGFRA* gene amplifications are among the most common genetic aberrations of receptor tyrosine kinases in GBM occurring in 57.4% and 13.1% of patients, respectively^1^. Despite this knowledge, targeted therapies have not worked well in GBM, which supports a tenet by which the most prominent oncogenic drivers might be required for tumor initiation, but certainly do not confer oncogenically addictive properties to GBMs. The current standard of care for GBM patients does not include precision medicine interventions but is based on alkylating cytotoxic agents (temozolomide), which show some benefit but to which resistance ultimately develops. Other classes of cytotoxic agents may therefore deserve re-evaluation given the difficulties observed with targeted therapies.

Microtubules are the target of many first-line anti-cancer drugs. These drugs are classified as microtubule stabilizers (taxanes, epothilones), destabilizers (eribulin), and vinca alkaloids (vinblastine (VB), vincristine), and have more complex effects^4,5^. Cells are sensitive to these drugs during mitosis, eliciting a defense mechanism called the spindle assembly checkpoint (SAC), which stalls mitosis until the insult has dissipated. In vitro, cells in prolonged SAC status either trigger apoptosis or reverse without cytokinesis, giving rise to senescent, micronucleated or multinucleated cells, and polyploidy^6,7^. The extent to which anti-mitotic actions can explain the clinical benefit of anti-microtubule drugs is hotly debated^8,9^. However, it is clear that perturbation of mitosis in cell culture reliably reports on the anti-microtubule activity of these drugs, thus serving as a useful surrogate for clinical activity.

Stathmin1 (STMN1) is a broadly expressed 17 kDa protein that binds to unpolymerized αβ-tubulin heterodimers. In normalcy, STMN1 inhibits microtubule polymerization by sequestering tubulin dimers, and triggering depolymerization events at plus ends^10^. Binding of STMN1 to tubulin is negatively regulated by serine phosphorylation on several sites, which are the substrates of multiple kinases^11,12^. Therefore, phosphorylation of STMN1 indirectly promotes tubulin polymerization and stabilizes microtubules. Although STMN1 is widely expressed and is an important negative regulator of tubulin, its functional role in cancer remains ill defined, especially with regards to anti-microtubule drug treatment efficacy.

To explore how microtubule inhibitors might be useful in GBM, we tested them in a new murine model of platelet-derived growth factor receptor-α (PDGFRα)-driven GBM and found that PDGFRα activity synergizes with the anti-microtubule actions of VB through STMN1 dephosphorylation events. Our results suggest a signaling axis that sensitizes cells to VB cytotoxic activities through STMN1 phosphorylation. The work described herein provides a roadmap for studies on phosphorylation of STMN1 and microtubule-targeting anti-cancer agents.

## Results

### Activation of PDGFRα requires p53 loss for tumorigenesis

**Fig. 1:**
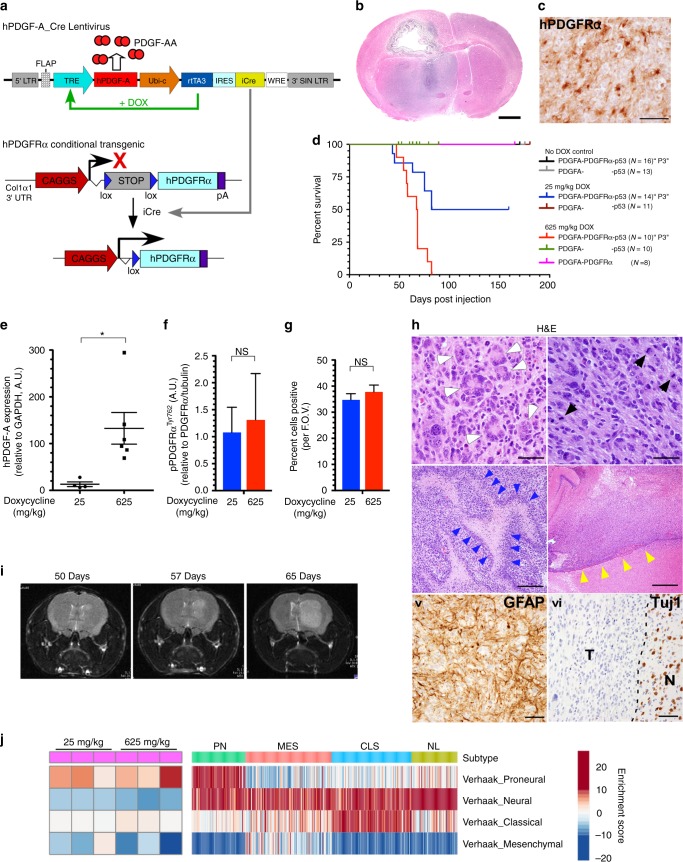
Spatiotemporal activation of PDGFRα in the CNS produces proneural GBM in adult mice. **a** Schematic of the conditional human PDGFRα cDNA transgene driven by the CAG promoter whose activity is prevented by a floxed stop cassette (LSL) until removed by Cre recombinase. The transgene was knocked into the 3′-UTR of the Col1α1 gene. **b** Representative photomicrograph of an H&E-stained FFPE section of a P3 brain tumor (scale bar, 1 mm). **c** Anti-hPDGFRα IHC (scale bar, 250 μm). **d** Tumor-free survival (Kaplan–Meier) analysis of three separate cohorts of mice of indicated genotypes fed the DOX diets. **e** qPCR analysis of PDGF-A mRNA from tumors of mice fed a 25 and 625 mg kg^−1^ DOX diet. *p < 0.03, (Student’s *t*-test). **f** Quantitative western blottings for phospho-PDGFRαTyr762 from lysates of P3 end-stage brain tumors (error bars denote SD; *n* = 3; NS, not significant by the Student’s *t*-test). **g** Percent of cells from end-stage tumors scored for proliferation using Ki67 marker by IHC on FFPE sections from P3 tumors from mice fed with 25 mg kg^−1^ and 625 mg kg^−1^ DOX diets. Average values of positive cells per field of view (FOV) (× 40). (error bars denote SD; *n* = 12 (4 FOVs per mouse); NS, not significant by the Student’s *t*-test). **h** P3 tumors have features of glioblastoma. Representative photomicrographs of FFPE tumor sections stained with H&E, IHC for GFAP and Tuj1 (NeuN) (scale bars, top and bottom row 50 μm, middle left 200 μm, middle right 500 μm). T, tumor; N, normal brain. **i** Photomicrographs of serial T2 flair MR imaging of a representative P3 GBM tumor. **j** Gene expression profiles of P3 GBM tumors from both low (25 mg kg^−1^ DOX) and high (625 mg kg^−1^ DOX) PDGF-A were used to perform GSEA against gene-set lists representing all four GBM subtypes. For scatter plots, the center line represents the mean and upper and lower lines SD

In GBM, overexpression and chronic activation of non-mutant, wild-type (WT) PDGFRα is the second most common genomic aberration of a receptor tyrosine kinase and 48% of these PDGFRα-positive GBMs are associated with loss-of-function mutations within the tumor suppressor gene *TP53*^1–3^. In fact, co-occurrence analysis of *PDGFRA* amplification/overexpression with other genes revealed that loss of *TP53* is the most statistically significant co-occurrence event (Supplementary Table 1). To understand how deregulated PDGFRα signaling leads to gliomagenesis, and to expose PDGFRα-dependent therapeutic vulnerabilities, we generated a conditional mutant mouse engineered to overexpress the human PDGFRα receptor in a Cre recombinase dependent manner (Fig. 1a and Supplementary Fig. 1a-c) (hereafter referred to as “P1” mice), which were crossed to a conditional loss of p53 function strain^13^ (hereafter referred to as “P2” mice for PDGFRα;p53). Exposure to Cre recombinase results in the excision of the Lox-Stop-Lox (LSL) cassette, expression of hPDGFRα, and deletion of p53.

In glioma, autocrine and paracrine expression of PDGF-A leads to chronic PDGFR signaling^14–16^. Therefore, to simulate this clinically relevant observation in P1 and P2 mice, we performed intracranial stereotactic injections of a lentivirus engineered to express an inducible (doxycycline, DOX) human PDGF-A complementary DNA ligand along with the constitutive expression of Cre in the striatum of adult (> 12 weeks) P1 and P2 mice. DOX administration induces expression of hPDGF-A and results in the activation of hPDGFRα kinase activity. Mice were fed ad libitum a diet containing DOX and were observed for the development of neurological signs of the central nervous system (CNS) neoplasm. PDGF-A;P animals on a DOX diet failed to develop brain lesions up to 165 days post injection (Fig. 1d), indicating that overexpression and activation of PDGFRα alone is insufficient for tumorigenesis in the CNS of mice. On the other hand, PDGF-A;P2 (hereafter called “P3”) mice when fed a DOX diet developed brain tumors that expressed human PDGFRα (Fig. 1b, c). These results demonstrate that our bipartite system, which offers a strict control over somatic, spatiotemporal overexpression, and activation of hPDGFRα kinase, requires loss of p53 function to initiate tumorigenesis in adult mouse CNS.

PDGF-A has two distinct C-terminal isoforms that result from alternative splicing^17^ giving rise to either a long (L) isoform that includes a positively charged extracellular matrix retention motif, or a short isoform (S) lacking this positively charged C-terminal, which is thought to diffuse more freely^18^. The activities of these isoforms are yet to be investigated in vivo. We determined using our P3 model that there are no functional differences between the two PDGF-A isoforms in tumor formation and histopathology (Supplementary Fig. 2). Moreover, in interrogating TCGA GBM database, we determined that PDGF-A(S) is overwhelmingly more expressed (97.8% of all PDGF-A transcripts) than its (L) counterpart (Supplementary Fig. 2), leading us to exclusively use hPDGF-A(S) in our studies.

### PDGF-A expression levels regulate tumorigenesis

We hypothesized that the wide range of PDGF-A expression levels observed in GBM patients (Supplementary Fig. 2f) have significant effects on PDGFRα signaling strength and form. In fact, comprehensive studies on the consequences of PDGF-A expression levels on gliomagenesis and signaling, and cancer in general, have never been rigorously addressed. Therefore, we set out to determine whether chronic but variable PDGF-A expression affects tumorigenesis. Cohorts of P3 mice were fed diets containing no DOX (control), low DOX (25 mg kg^−1^), and high DOX levels (625 mg kg^−1^), and followed for survival. We found that control (no DOX) P3 mice did not develop intracranial tumors upon necropsy after 170 days post injection at which point the experiment was terminated, suggesting that overexpression of non-ligand stimulated PDGFRα in the context of p53 nullizygocity is insufficient for tumorigenesis. However, 100% of the high DOX P3 mice succumbed from intracranial tumors and roughly half of the low DOX P3 animals died of brain tumors with median survivals of 67.5 days vs. 131 days, respectively (Fig. 1d) (*P* = 0.0037 log-rank (Mantel–Cox) test)). PDGF-A expression in the context of p53 nullizygocity failed to initiate tumorigenesis during a 150-day period, after which point the experiment was terminated (Fig. 1d). It is known that ectopic CNS expression of PDGF-A in mice elicits a dose-dependent proliferation of PDGFRα-positive oligodendrocyte precursor cells (OPCs)^19–21^. Similarly, we observed that hPDGF-A;p53^−/−^ mice fed a high-DOX but not a low-DOX diet displayed a hyperproliferation of OPCs (Supplementary Fig. 3).

To substantiate the generation of graded PDGF-A expression levels in the tumors from the low- and high-DOX P3 animals, we performed quantitative reverse-transcription PCR for human PDGF-A on RNA isolated from their tumors and showed significantly higher levels of PDGF-A transcripts in high- vs. low-DOX P3 tumors (Fig. 1e). Surprisingly, these higher levels of hPDGF-A expression did not correspond to significant increases in phospho-PDGFRα levels when compared with low-DOX tumors when measured by western blotting (Fig. 1f). Consistent with this finding, the Ki67 proliferative index was similar in high- and low-DOX P3 tumors (Fig. 1g). Together, these findings highlight the robustness of our model system to generate a DOX-dependent graded levels of PDGF-A expression in vivo that engage receptor kinase activity in tumor cells.

We found that the markedly reduced survival penetrance of the low-DOX P3 cohort was not attributed to a deficient recombination of the (LSL) cassette, as identical levels of hPDGFRα expression was observed in the brains of low- or high-DOX-treated P3 animals 20 days post injection (Supplementary Fig. 4a–d). Surprisingly, we noticed the presence of persisting clusters of hPDGFRα-positive cells in the brains of the surviving P3 animals 150 days post injection. Although the number of which and their level of hPDGFRα expression were similar to those observed in P3 mice 20 days post injection, expression of hPDGF-A, phospho-hPDGFRα, and consequently their proliferative indexes were significantly reduced when compared with those at 20 days post injection (Supplementary Fig. 4e–h). This demonstrates that in half of the P3 mice on low-DOX diets, loss of hPDGF-A expression resulted in a growth arrest of hPDGFRα-positive clusters after 20 days post injection. Given the oligoclonal nature of tumor initiation in our system, an en masse tumor cell-centric attenuation of DOX-inducibility is unlikely to account for suppression of PDGF-A expression but rather may be reflective of negative microenvironmental pressures exerted on tumor cells. In fact, we cannot rule out a pro-tumorigenic contribution of OPCs in the high-DOX P3 cohort.

Collectively, these results demonstrate that every P2 animal injected with the lenti-hPDGF-A-Cre virus developed hPDGFRα-positive lesions, and that hPDGF-A expression and hPDGFRα activity in the context of p53 nullizygocity is necessary to initiate tumorigenesis and to sustain growth since half of tumor-bearing animals that failed to maintain PDGF-A expression did not develop lethal tumors.

### P3 tumors exhibit features of proneural GBM

The P3 tumors displayed features that are consistent with high-grade gliomas. Tumors were highly cellular, containing poorly differentiated cells with marked nuclear atypia, multiple mitoses (Fig. 1h, black arrowheads), giant multinucleated cells (Fig. 1h, white arrowheads), and areas of pseudopalisading necrosis (Fig. 1h, blue arrowheads). P3 tumors were highly infiltrative, lining up and accumulating against host brain structures, forming secondary structures of Scherer (Fig. 1h, yellow arrowheads). The tumor cells expressed variable levels of the astrocytic marker glial fibrillary acidic protein (GFAP), and expression of the neuronal marker NeuN was absent (Fig. 1h). These histopathological features are consistent with those observed in GBM. Primary or de novo GBMs are explosively growing tumors that remain asymptomatic until clinical manifestations. We used magnetic resonance imaging (MRI) to detect and longitudinally quantify volumetric tumor growth over time in P3 mice. Tumors displayed rapid changes in T2 flair signals corresponding to brisk volumetric increases (Fig. 1i and Supplementary Fig. 1d). The expansion in tumor volumes was not initially accompanied with clinical manifestations such as weight loss, lack of grooming behavior, lack of nesting, hunch posture, and reduced ambulation, an observation consistent with clinical presentation of GBM.

Finally, we analyzed the transcriptome of the P3 GBM tumors and performed a gene-set enrichment analysis using gene-set lists defining the four human GBM subtypes^1,3^. Both low- and high-PDGF-A-expressing P3 GBMs significantly associated with the human Proneural subtype (Fig. 1j). Lastly, the hPDGF-A messenger RNA expression levels in low- and high-DOX P3 GBMs were comparable to and well within the physiologically relevant range displayed in human GBMs (Supplementary Fig. 2f). We also compared the hPDGFRα transgene transcript levels with those of human GBMs and observed robust receptor mRNA expression, similar to that found in the top tenth percentile of GBM tumors (Supplementary Fig. 1e).

Taken together, this new model of hPDGFRα-driven tumorigenesis recapitulates several salient features of GBM biology including chronic autocrine and paracrine stimulation of overexpressed PDGFRα with clinical presentations that are consistent with those observed in GBM patients. These results demonstrate that constitutive hPDGF-A expression combined with high hPDGFRα overexpression can trigger gliomagenesis in vivo in a p53-dependent manner. The high-grade gliomas derived have histopathological and molecular features reminiscent to those observed in human proneural GBMs. Our results suggest that cellular responses to hPDGF-A stimulation are both ligand and receptor-level dependent. To better define this observation mechanistically, we generated primary cell cultures from our GBM tumors in order to study the effects of variable ligand stimulation on receptor activation, signaling events, and cellular behaviors.

### Graded PDGFRα activation promotes distinct signaling outcomes

Acute, ligand-saturating stimulus of PDGFR signaling events are well characterized^18,22^. However, comparative studies on signal transduction pathways originating from weak vs. robust, chronically activated PDGFRs that are much more cancer relevant, have never been performed. We analyzed signaling pathways from chronically activated PDGFRα in primary cell cultures isolated from P3 GBM tumors with the goal to identify PDGFRα activation-dependent therapeutic vulnerabilities. Using primary cells derived from P3 tumors that retained (1) high expression of hPDGFRα and (2) the capacity to express hPDGF-A and activate hPDGFRα kinase activity in a DOX-dependent manner (Supplementary Fig. 5), we performed unbiased and global comprehensive quantitative proteomic and phospho-proteomic (pTyr, pSer, and pThr) analyses of chronic, low (0.1 μg mL^−1^ DOX 48 h), and high (10 μg mL^−1^ DOX 48 h) hPDGF-A stimulation in comparison with control, unstimulated cells. We utilized isobaric label-based quantitative mass spectrometry (MS) to measure expression of proteins and phospho-isoforms^23,24^. This analysis yielded expression data for 7528 proteins across all conditions. To measure the alteration of signaling pathways upon treatment, we performed successive phosphopeptide enrichment and pTyr immunopurification to quantify 5849 phosphopeptides (of which 195 contained a tyrosine phosphorylation event) belonging to 1871 unique proteins. As protein expression can influence measurements of enriched posttranslation modifications, we normalized quantitative values for phosphorylation sites to protein-level expression measured in un-enriched samples and calculated differences between stimulated and control as Log_2_ fold change (FC) for each phosphopeptide.

**Fig. 2:**
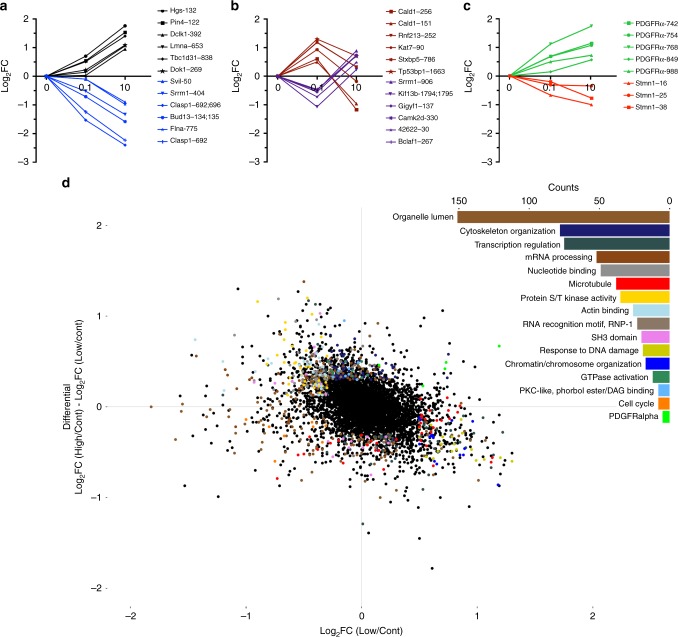
Differential chronic signaling from weakly and strongly activated hPDGFRα. **a**–**c** Log_2_ fold change of representative phospho-sites from quantitative phospho-proteomics dataset. **d** Global quantitative MS/MS phospho-proteomic analysis of low and high chronic hPDGFRα activation. Plotted are the Log_2_ fold change (FC) of low hPDGFRα activation over unstimulated hPDGFRα for 5849 normalized phosphopeptides (*x* axis) against the differential between Log_2_FC high vs. control and Log_2_FC low vs. control hPDGFRα activation (*y* axis). Top right inset, 15 top scoring GO categories of outlier (Log_2_FC < − 0.5, > + 0.5) phospho-events. Green data points are hPDGFRα autophosphorylation sites. Results are from single measurements of pooled biological triplicates

As expected, the levels of many phospho-sites followed incremental or decremental changes upon graded PDGF-A stimulation (Fig. 2a). The PDGFRα autophosphorylation sites Y742, Y754, Y762, Y849, and Y988 followed an incremental trend (Fig. 2c). Unexpectedly, however, we observed a significant trend reversal for a substantial numbers of phosphopeptides. For example, low ligand engagement of hPDGFRα led to increases in levels of phosphorylation for specific sites, whereas high ligand engagement of receptor resulted in decreases for those same phospho-sites (Fig. 2b). The converse effect was also observed for a number of phospho-sites (Fig. 2b)

This demonstrated that chronic activation of hPDGFRα leads to complex dynamics of signaling pathways that are heavily influenced by the strength of the receptor activation. When analyzed globally, the majority (~80%) of the phosphopeptides display minimal changes (Log_2_FC between − 0.5 and 0.5) between control, low, and high ligand stimulation; however, ~20% showed significant variations between low and high hPDGF-A stimulation (Fig. 2d). To gain insight into potential functionalities of these events, we performed Gene Ontology term enrichment analysis on proteins from outlier phosphorylation events (Log_2_FC < − 0.5, > + 0.5) (Fig. 2d, upper right inset). Of the top-ranking categories are proteins associated with microtubule biology and of particular interest are several phosphorylation sites of the tubulin-binding protein STMN1 (Fig. 2c). These are relevant, because (1) there are known biological functions that have been associated with phosphorylation at these sites and (2) microtubule disruptors are potent chemotherapeutic agents.

### PDGFRα-STMN1 axis confers VB sensitivity

**Fig. 3:**
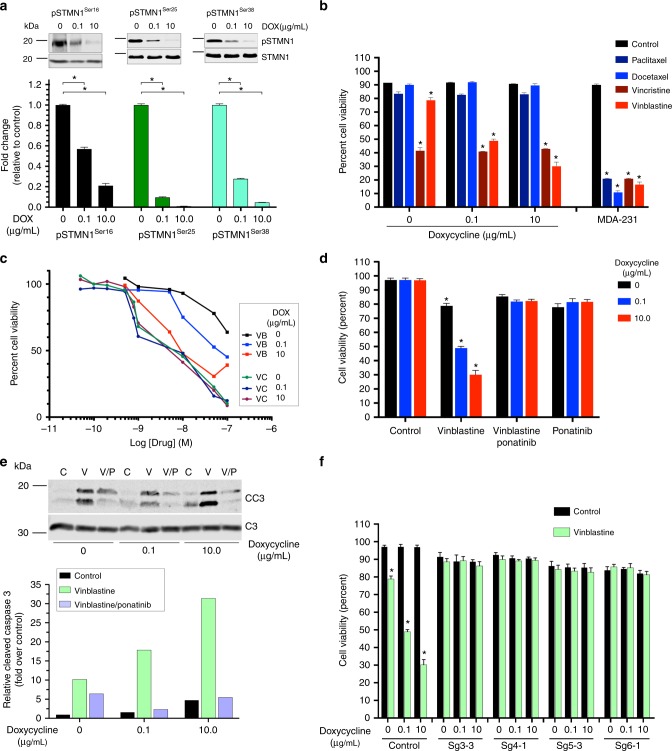
hPDGFRα-STMN1 signaling axis confers sensitivity to vinblastine. **a** Phospho-STMN1 Ser16, Ser25, and Ser38 levels decrease in a dose-dependent manner. Representative images and quantitation of phospho-STMN1 Ser16, Ser25, and Ser38 immunoblots (error bars denote SD; *n* = 3, *p < 0.0001, by Student’s *t*-test when compared with 0 DOX controls). **b** PDGFRα-positive GBM cell sensitivity to microtubule drugs. Cells were treated with the indicated drugs and viability assessed via trypan blue assays. MDA-231 breast carcinoma cell line serves as a positive control for drug sensitivities. Cells were treated with docetaxel and paclitaxel (500 nM, 96 h, MDA-231 cells were treated with 100 nM), vinblastine (100 nM, 24 h), and vincristine (200 pM, 24 h). Cells were grown in the absence or presence of activated hPDGFRα (error bars denote SD; *n* = 3, *p < 0.0001 by Student’s *t*-test when compared with 0 DOX control). **c** Synthetic decreases in cell viability between hPDGFRα activity and vinblastine treatment. Dose-response curves of vinblastine and vincristine in the presence and absence of PDGFRα activity. **d** Inhibition of hPDGFRα activity using ponatinib (1 μM) blocks the synthetic sensitivity between hPDGFRα and vinblastine (error bars denote SD; *n* = 4, *p < 0.0001 by Student’s *t*-test when compared with 0 DOX control). **e** Synthetic induction of apoptosis in vinblastine and hPDGFRα active cells. Quantitative western blotting of cleaved and total caspase 3 of GBM cells treated for 24 h with vinblastine (100 nM), in the absence or presence of PDGF-A with and without the PDGFR inhibitor ponatinib (1 μM) (error bars denote SD; *n* = 3). **f** The hPDGFRα-vinblastine synthetic lethal effect is mediated through STMN1. STMN1 CRISPR/Cas9 knockout abrogates the heightened lethal outcome of hPDGFRα activity and vinblastine. Representative clonal cultures from four independent sgRNAs against STMN1 are shown (error bars denote SD; *n* = 4, *p < 0.0001 by Student’s *t*-test when compared withto 0 DOX control)

We observed three phospho-sites in STMN1 (Ser16, Ser25, and Ser38) that were inversely regulated by hPDGFRα activity in our phospho-proteomics dataset (Fig. 2c). Validation by quantitative western blotting demonstrated that an increase in hPDGFRα activity indeed led to a significant decrease in the levels of phospho-STMN1 Ser16, Ser25, and Ser38 (Fig. 3a). Given STMN1’s role in microtubule dynamics, we hypothesized that a graded engagement of hPDGFRα activity might sensitize cells to microtubule assembly inhibitors in an STMN1-dependent manner. To this end, we first determined that the hPDGFRα cells are sensitive to the vinca alkaloids vincristine and VB but resistant to the taxanes docetaxel and paclitaxel (Fig. 3b). VB treatment demonstrated a reproducible hPDGFRα activity-dependent cytotoxic effect, whereas vincristine induced a cytotoxic effect regardless of hPDGFRα activity (Fig. 3b). In fact, activation of hPDGFRα led to an increase in the cytotoxic sensitivity of these cells to VB in a dose-dependent manner (Fig. 3c). However, this effect was not observed with vincristine (Fig. 3c) and treatment with paclitaxel and docetaxel did not elicit cytotoxic activities regardless of hPDGFRα activation status (Supplementary Fig. 6a).

The hPDGFRα activity-mediated increase in sensitivity to VB was completely eliminated upon inhibition of hPDGFRα with ponatinib (Fig. 3d) and the reduction in cell viability observed resulted from induction of caspase-mediated apoptosis (Fig. 3e), which was also eliminated by ponatinib treatment. The reduction of the levels of phospho-STMN1 Ser16 upon PDGFRα activity was also eliminated by treatment with other PDGFR inhibitors, thus demonstrating specificity of PDGFRα for phospho-STMN1 Ser16 (Supplementary Fig. 6b). Finally, CRISPR-mediated STMN1 knockout revealed that the synthetic lethality of VB and hPDGFRα activity is mediated by STMN1 (Fig. 3f and Supplementary Fig. 6c-e). It is noteworthy that STMN1 knockout GBM cells did not acquire sensitization to taxanes (Supplementary Fig. 6f), contrary to what has been observed in breast cancer^25,26^, and DOX treatment does not induce a cellular stress response that accounts for STMN1 dephosphorylation, as PDGFRα inhibition prevents decrease in phospho-STMN1 Ser16 levels (Supplementary Fig. 6g). These results demonstrated an absolute requisite for STMN1 in the hPDGFRα synthetic VB-mediated cytotoxicity.

Interrogating the GBM TCGA database for an association between PDGFRα and STMN1 expression, we discovered that STMN1 is significantly co-expressed with PDGFRα but not with other important GBM drivers such as epidermal growth factor receptor (EGFR) (Supplementary Fig. 7a). In fact, EGFR activity in EGFR-driven mouse GBM cell cultures^27–29^ had no effect on the levels of phospho-STMN1 Ser16 and on cell viability in response to VB treatment (Supplementary Fig. 7b). Taken together, these results demonstrate that PDGFRα stimulation result in a decrease in phospho-STMN1 Ser16 levels, and that VB treatment is synthetic lethal to a PDGFRα-STMN1 signaling axis.

### Exacerbation of VB-triggered mitotic apoptosis by PDGFRα

**Fig. 4:**
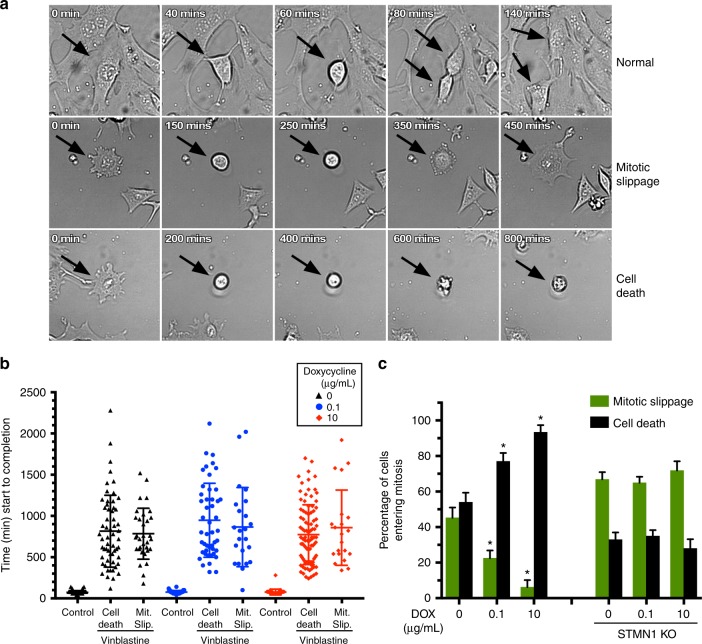
hPDGFRα-STMN1 signaling exacerbates vinblastine toxicity during mitosis. **a** Representative photomicrographs of phase-contrast time-lapse microscopy of cells untreated or treated with vinblastine. In the absence of vinblastine cells entering mitosis proceed to divide normally within an average of ~120 min, whereas in the presence of vinblastine cells entering mitosis undergo arrest followed by either apoptosis or mitotic slippage. **b** Quantification of time spent in mitosis from **a**. **c** hPDGFRα activity promotes cell death in vinblastine during mitosis in an STMN1-dependent manner. Percentage of cells entering mitosis that undergo slippage or apoptosis for parental and STMN1 CRISPR KO cells (error bars SD; *n* = 24, *p < 0.0001 by Student’s *t*-test, when compared with no DOX control). For scatter plots, the center line represents the mean and upper and lower lines SD

Cells in culture entering mitosis are particularly sensitive to microtubule-binding drugs. VB-poisoned microtubules activate the SAC, which is a persisting protective mechanism that induces cell cycle arrest during mitosis until the triggering defects are resolved through either (1) apoptotic cell death or (2) mitotic slippage, i.e., cells exiting the prolonged mitotic arrest and skipping cytokinesis^30^. We performed time-lapse microscopy on VB-treated P3 cultures in the presence and absence of activation of the PDGFRα-STMN1 axis to gain mechanistic insight into the synthetic lethality between PDGFRα and VB. This approach allows scoring of drug effects on division and death without the need for perturbing synchronization protocols and accounts for cell-to-cell variation^31^. Cells were treated with VB with and without hPDGFRα activation and imaged for 48 h (Fig. 4a and Supplementary Movie 1). We determined that the amount of time the cells spent in mitosis was drastically lengthened upon VB treatment (Fig. 4b) and that hPDGFRα activation does not increase the proportion of cells entering mitosis (Supplementary Fig. 8a) nor does it affect the length of time cells spend in mitosis (Fig. 4b and Supplementary Fig. 8b). Consistent with those results, hPDGFRα activation did not increase overall cell growth (Supplementary Fig. 8c) despite a small increase in the proportion of cells in S phase of the cell cycle (Supplementary Fig. 8b). VB treatment caused mitotic defects independent of hPDGFRα activation. Of the cells entering mitosis, 45.24% underwent mitotic slippage and 54.05% underwent apoptosis (Fig. 4c). However, most cells died during mitotic arrest when hPDGFRα was activated, whereas most exited mitosis without death when hPDGFRα was not activated (Fig. 4c). Importantly, this synthetic lethal interaction between hPDGFRα activity and VB was STMN1-dependent since STMN1 KO cells are no longer subject to hPDGFRα activity (Fig. 4c).

Cells undergoing mitotic slippage are typically polyploid. We assessed DNA content analysis by flow cytometry and confirmed that treatment of P3 cultures with VB resulted in cells with higher DNA content, a sign of polyploidy (Supplementary Fig. 9a). The fate of cells that are undergoing mitotic slippage can be varied. Cells can enter a state of senescence, develop micronuclei, or become multinucleated. We detected a significant increase in the number of micronucleated cells but no increase in multinucleated cells in cultures treated with VB (Supplementary Fig. 9b). However, we cannot rule out senescence as an outcome of mitotic slippage. Finally, VB can also cause apoptosis independently of mitosis in interphase cancer cells^32^. To determine the extent of interphase apoptosis, VB-treated cells were labeled with the tubulin-binding fluorescent dye SiR-tubulin (Spirochrome) and time-lapse imaged for 8 h. Mitosis-independent apoptosis was scored as the length of time spent between degradation of the nuclear envelope and death. We did not observe cell death outside of mitosis in VB-treated cells (Supplementary Fig. 9c, d). Taken together, our results show that hPDGFRα-STMN1-dependent cytotoxic sensitization to VB occurs mainly during mitosis.

### The PDGFRα-STMN1 axis sensitizes GBM to VB treatment in mice

**Fig. 5:**
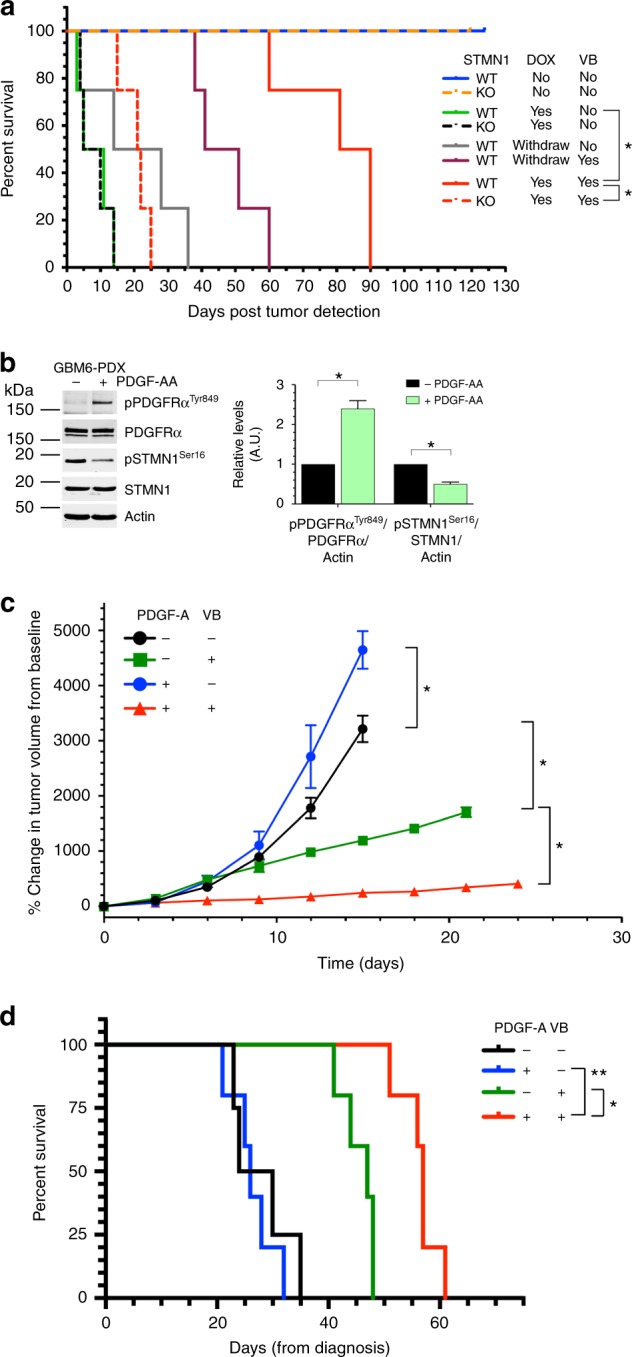
Prolonged survival with vinblastine in GBM is PDGFRα activity and STMN1 dependent. **a** Tumor-free survival (Kaplan–Meier) analysis of mice intracranially injected with hPDGFRα GBM cells wild-type or null for STMN1 fed a DOX diet (625 mg kg^−1^). Upon tumor detection (as determined by an 8–10% weight loss), mice were enrolled randomly on control or vinblastine (0.5 mg kg^−1^ I.P. every 3 days) until moribund. Cohorts of mice were also withdrawn from DOX at tumor detection. *p = 0.0067 (log-rank Mantel–Cox test). **b** PDGFRα-activity-dependent decrease in levels of phospho-STMN1 Ser16 in the human GBM6 PDX line. Shown are representative western blotting of the indicated antibodies and graphical representation of quantitative measurements from biological triplicates. *p < 0.0001 by Student *t*-test. **c** Human GBM PDX sensitized to vinblastine treatment in vivo by chronic activation of PDGFRα. Tumor volumes of the GBM6 PDX line genetically modified to express human PDGF-A ligand in a DOX-inducible manner grown in flanks of immunocompromised Ncr^Nu/Nu^ mice. Tumor response is expressed as the percentage change from the baseline tumor volumes at the time of treatment initiation (~100 mm^3^, Day 0). Baseline tumor volumes are listed in Methods. *n* = 5, *p < 0.0001 by Student *t*-test. **d** Tumor-free survival (Kaplan–Meier) analysis of mice intracranially injected with human GBM6 PDX cells from **c**. Upon tumor detection (as determined by an 8–10% weight loss), mice were enrolled randomly on control or vinblastine (0.5 mg kg^−1^ IP every 3 days) until moribund. *p = 0.0027, **p = 0.018 (log-rank Mantel–Cox test)

Having demonstrated a sensitization of VB efficacy upon hPDGFRα-STMN1 stimulation in P3 cultures, we validated this observation in our pre-clinical model in vivo. We implanted parental and STMN1 knockout P3 GBM cells intracranially in immunodeficient mice that were fed a DOX diet and monitored tumor growth by daily weighing until a 8–10% loss was observed at which point animals were randomly enrolled in control or VB treatment cohorts (Fig. 5a). P3 GBM cell-bearing mice (parental and STMN1 knockout derivatives) failed to develop tumors in the absence of DOX. P3 STMN1 knockout GBM cells developed tumors at the same rate as STMN1 WT parental cells when mice were fed a DOX diet. Treatment of hPDGFRα-bearing GBM mice with VB significantly prolonged survival when compared with untreated mice (median survival 85.5 days vs. 8 days, respectively, *P* = 0.0067 log-rank Mantel–Cox test). This increase in survival was drastically diminished in animals with P3 GBMs that were null for STMN1 (median survival 21.5 days, *P* = 0.0067 log-rank Mantel–Cox test). Moreover, the requirement for PDGFRα activity for the observed heightened VB sensitivity was best exemplified when DOX was withdrawn upon tumor detection and treated with VB, resulting in a shorter median survival (46 days). Overall, VB cytotoxic therapeutic effects observed in P3 GBMs in vivo are greatly enhanced upon PDGFRα activation and are dependent on STMN1.

We validated these observations using a human GBM PDX^33^. First, similar to our mouse GBMs, PDGF-AA activation of PDGFRα in the GBM6 PDX decreased the levels of phospho-STMN1 Ser16 (Fig. 5b) and sensitized the cells in vitro to VB (Supplementary Fig. 10). We further demonstrated this observation in vivo by engineering the GBM6 PDX with a lentivector to express a DOX-inducible hPDGF-A ligand. Intracranial and flank implantation of these cells in immunocompromised mice led to tumor growth and an acquired VB sensitivity only in the context of PDGFRα activation, which considerably reduced tumor growth and extended survival (Fig. 5c, d).

## Discussion

We developed a new genetically engineered model of Proneural GBM based on chronic, autocrine hPDGF-A stimulation of overexpressed hPDGFRα in the context of loss of p53, genetic events that are clinically supported by TCGA data^1^. Using a tetracycline-inducible lentiviral system, we demonstrated that graded hPDGF-A engagement of hPDGFRα led to activation of unique signaling pathways that exposed an STMN1-mediated therapeutic vulnerability to VB.

Our new model revealed important aspects of gliomagenesis that were previously unknown. First, overexpression of PDGFRα alone is insufficient for tumor formation, but rather requires ligand activation. We previously have reported the temporal sequence of genetic events in glioma development using mathematical modeling and computational analysis of TCGA data and shown that gain of chromosome 7 associated with elevated expression of PDGF-A (located on chr7) are common early genetic events^34^. Keeping with these observations, we demonstrated an absolute requirement for hPDGF-A expression for gliomagenesis in our model. Second, there appears to be a requisite for loss of a tumor suppressor gene function (e.g., p53) for hPDGFRα-driven tumorigenesis in mice, as activation of PDGFRα alone in the absence of p53 loss does not result in tumorigenesis. Mechanistically, activation of oncogenic hPDGFRα in vivo likely triggers a senescence program, which is counteracted by the loss of p53. Abrogation of p53 function is a common event in GBM that is often associated with PDGFRα amplification but never with EGFR amplification^1,3^. This nearly uniform mutual exclusiveness suggests a fundamental difference in either cell type-specific requirements or signaling emanating from these receptors for selective loss of p53 function during gliomagenesis.

An important advancement from our work is the analysis of signaling under chronic, low- and high-level receptor activation using innovative proteomics. Most previous studies of PDGFR signaling relied on acute, saturating receptor stimulation. Although informative, this approach is far from clinical reality. Here we demonstrated that hPDGFRα signaling pathway wiring is dependent on the intensity of activation of the receptor, and that chronic, low activation versus high activation revealed differences that we exploited therapeutically.

**Fig. 6:**
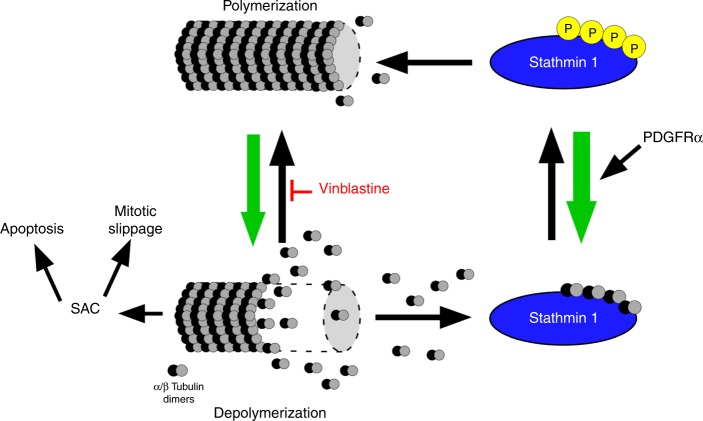
PDGFRα and STMN1 cooperate to exacerbate the cytotoxic effects of vinblastine. Cartoon depicting the mechanism by which PDGFRα, through STMN1 dephosphorylation, leads to enhanced microtubule depolymerization. Vinblastine-poisoned microtubules undergo reduced polymerization at the + end effectively resulting in depolymerization, which during mitosis, triggers SAC and results in either apoptosis or mitotic slippage

The most significant aspect of our study is our finding that engaging the kinase activity of hPDGFRα, and the ensuing dephosphorylation of STMN1, selectively sensitizes cells to killing by VB (Fig. 6). Specifically, we observed a shift in the equilibrium of the cellular responses to VB treatment between mitotic arrest-mediated cell death and mitotic slippage, favoring cell death (Fig. 4). The amount of time spent in mitosis upon VB treatment was not changed by hPDGFRα activity, and this outcome was STMN1 dependent. It is possible that cyclinB/Cdk1 complexes are involved given that they have been proposed to be responsible for the execution of the apoptotic phenotype upon SAC activation^30^ and that Cdk1 is known to phosphorylate STMN1^11^. Activation of hPDGFRα may lead to a reduction in Cdk1 activity, a nodal point that controls two pathways that are affected by VB. Alternatively, mis-regulation of STMN1 may sensitize microtubule dynamics to inhibition by VB through changes in the formation of tubulin_2_.STMN1.VB complexes^35^. It is also possible that phosphorylation of STMN1 at Ser16 interferes with VB binding (but not vincristine) to the hydrophobic patch of α tubulin thus reducing VB affinity and biological activity, an effect that is reversed by dephosphorylating STMN1. In fact, the levels of phospho-STMN1 in cancer cells may serve as a biomarker of VB sensitivity.

Another important finding from our study was the sensitization of low phospho-STMN1, hPDGFRα-positive, but not EGFR-positive, GBM cells to VB. In addition, the specificity of VB over vincristine for the observed hPDGFRα synthetic lethal interaction is remarkable given the similar structure and biochemical actions of the two drugs. One possible explanation lies in the detailed structure of the ternary complex that forms between two tubulin dimers, one molecule of STMN1, and one of VB. This complex was first demonstrated in solution using biophysical analysis that showed potentiation of VB binding to tubulin by STMN1^35^. Subsequent X-ray crystallographic studies of the same complex showed that VB binds at the interface between the two tubulin dimers^36^. We suspect that formation of this tetrameric complex causes STMN1 to promote cell killing by VB, and that different substitutions onto the vinca core in vincristine compared to VB impedes formation of the tetrameric complex.

Using genetically accurate models of Proneural GBM, we revealed that the cytotoxic effects of an important chemotherapeutic drug are drastically enhanced in a signaling-specific context. Our work demonstrates that STMN1 phospho-levels can enhanced the efficacy of an anti-cancer molecule that was not, until now, considered to be practical for GBM^37^. The data presented here have clinical potential and provide a rationale for future development of better brain penetrating anti-microtubule cancer therapeutics for GBM.

## Methods

### PDGFRα conditional mice, procedures, and genotyping

All mouse procedures were performed in accordance with Beth Israel Deaconess Medical Center recommendations for the care and use of animals, and were maintained and handled under protocols approved by the Institutional Animal Care and Use Committee. A detailed description of the strategy to generate the targeted knockin of the CAG-floxed stop cassette PDGFRα cDNA minigene into the mouse collagen1α1 gene locus is included in Supplementary Methods. The CAG-LSL-PDGFRα mice were mated to the conditional p53^2lox^^13^ mice to generate compound strains. Activation of PDGFRα expression and concomitant inactivation of *Tp53* in the brain was accomplished by stereotactic intracranial injections of the lenti hPDGF-A-Cre virus (Supplementary Methods and ref.^38^). Details on the cloning, production and purification of the virus are described in Supplementary Methods. Genotyping of the CAG-LSL-PDGFRα mice is performed from genomic DNA isolated from tail biopsies by PCR using oligonucleotide primers listed in Supplementary Methods.

### Histology and immunohistochemistry

Tumor-bearing brains were processed for formalin-fixed paraffin-embedded as follows: brains were excised and rinsed in phosphate-buffered saline (PBS) and serial coronal sections cut using a brain mold. Half of the sections were used to isolate primary cultures of tumor cells as described in Supplementary Methods and the other half were postfixed in 4% paraformaldehyde, embedded in paraffin, sectioned (5–10 μm), and stained with hematoxylin and eosin. For immunohistochemistry, sections were deparaffinized and rehydrated followed by antigen target retrieval and processing as described in Supplementary Methods. All antibodies (listed in Supplementary Methods) were diluted in blocking solution and immunobinding of primary antibodies was detected by biotin-conjugated secondary antibodies and Vectastain ABC Kit (Vector Labs, Inc.) using DAB (Vector Labs, Inc.) as a substrate for peroxidase activity, and counterstained with hematoxylin as described in the manufacturer’s protocol.

### Primary cell cultures

Primary cultures of tumors were established as follows: tumors were excised and minced in 0.25% trypsin (wt/vol),1 mM EDTA, and allowed to disaggregate for 15 min at 37 °C. The resulting cell suspension was then strained through a 70 μm cell strainer (Falcon). The single suspension of cells was washed in PBS twice and plated on 0.2% gelatin-coated tissue culture plates. Cells were fed every 24 h with fresh media that consisted of Dulbecco’s modified Eagle’s medium (DMEM) supplemented with 10% heat-inactivated fetal bovine serum (FBS) and antibiotics. The primary cultures of astrocytes were routinely stained for markers of astrocytic lineages by immunofluorescence to determine the purity of individual preparations.

### Immunoblotting and antibodies

Procedures describing western blotting and a detailed list of the antibodies used in these studies can be found in Supplementary Methods.

### Magnetic resonance imaging

MRI imaging was performed on a 1T Bruker DICON MRI scanner. Multislice *T*_2_-weighted spin-echo (echo time = 84.0 ms; repetition time = 2500 ms; field of view (FOV) = 20 mm; matrix = 160 × 160; 5 slices; slice thickness = 1 mm; total imaging time = 20 min). Images were acquired for each animal every 7 days during tumor development. Volumetric analysis for each tumor was conducted using Image J image processing software.

### RNA isolation and gene expression analyses

Total RNA was isolated from flash frozen tumors freshly excised from mice using Qiagen RNeasy RNA Isolation Kit. Gene expression analysis was conducted using the GeneChip® Mouse 2.0 ST Array (Affymetrix) at the Yale Center for Genome Analysis.

### Proteomic and phosphoproteomic sample preparation

Cell pellets were collected as pooled biological triplicates and then lysed with a buffer containing 50 mM HEPES (pH 8.5), 8 M urea, 150 mM NaCl, protease inhibitors (mini-Complete EDTA-free, Roche), and phosphatase inhibitors (PhosSTOP, Roche). Using a syringe, cells were passed through a 22-gauge needle 15 times for mechanical lysis. Lysates were cleared through centrifugation lysate and the protein concentration was determined using a BCA assay (ThermoFisher Scientific). Equal amounts of protein (4 mg) was reduced for 45 min at 37 °C with 5 mM dithiothreitol (DTT), alkylated with 15 mM IAA for 30 min at room temperature in the dark, before final reduction with 5 mM DTT for 15 min at room temperature. Protein content was then extracted through methanol–chloroform precipitation, before re-suspension in 50 mM HEPES, 8 M urea, and 150 mM NaCl. For proteolytic digestion, LysC (Wako, Japan) was added at a substrate:enzyme ratio of 100:1 and incubated for 3 h at 37 °C. Samples were then diluted to 1.5 mM Urea with 50 mM HEPES and digest overnight with Trypsin at room temperature with a substrate:enzyme ratio of 50:1. The peptide solutions were then acidified before solid-phase extraction via SepPak (Waters). Peptide samples were re-suspended in 1 mL 50% ACN, 2 M lactic acid, and 100 µg of each sample was removed, desalted, and saved for protein-level measurements. Phosphopeptide enrichment was performed according to ref.^39^.

Non-phosphorylated peptides saved prior to enrichment and enriched phosphopeptides were then suspended in 100 µL of 200 mM EPPS pH 8.5 before the addition of 30 µL of anhydrous acetonitrile, and 10 µL of a 20 µg/µL stock of TMT reagent. Samples were incubated for 1 h at room temperature before the addition of 10 µL 5% hydroxylamine. A small portion of each sample was mixed, desalted, and analyzed, to determine relative analyte abundance in each sample. The remaining sample was then mixed to ensure equal loading of peptide and phosphopeptide content and acidified before solid-phase extraction via SepPak. Following isobaric labeling, enriched phosphopeptides were enriched again for phospho-tyrosine (pTyr)-containing peptides. Enriched phosphopeptides were re-suspended in 450 µL of immuno-affinity purification (IAP) buffer (50 mM MOPS/NaOH pH 7.2, 10 mM Na_2_PO_4_, and 50 mM NaCl). A pTyr-specific antibody (P-Tyr-1000, Cell Signaling Technology) was incubated with protein A agarose beads (Roche) overnight at 4 °C in 1% PBS to bind the antibody to the beads. Subsequently, the antibody–bead mixture was washed 3 × with IAP before incubation with enriched phosphopeptides for 1 h at room temperature, to enable capture of pTyr-containing peptides. The supernatant, containing enriched phosphopeptides, was removed, desalted using a SepPak, and saved for offline fractionation. The beads were washed 1 × with IAP and 1 × with H_2_0 before performing two elutions using 75 µL of 100 mM formic acid. Enriched pTyr peptides were desalted and re-suspended in 1% formic acid before nanoflow liquid chromatography-tandem MS (nLC-MS/MS) analysis.

Non-phosphorylated and phosphorylated peptides were fractionated via basic-pH reversed-phase LC^40^. Non-phosphorylated samples were re-suspended in 5% ACN, 1% formic acid, and phosphorylated peptides were re-suspended in 1% formic acid before nLC-MS/MS analysis.

### MS analysis

MS analyses were performed on an Orbitrap Fusion Lumos mass spectrometer (ThermoFisher Scientific) coupled to an Easy-nLC 1200 ultra-high-pressure LC pump (ThermoFisher Scientific). Peptides were separated at 300 nL/min using an analytical column (75 µm inner diameter) that was packed self-packed with 0.5 cm of Magic C18 resin (5 µm, 100 Å, Michrom Bioresources) followed by 35 cm of Sepax Technologies GP-C18 resin (1.8 µm, 120 Å). LC buffers consisted of 0.1% formic acid (buffer A) and 80% ACN with 0.1% formic acid, and LC gradients were optimized to ensure equal elution of peptides throughout the analysis. Survey scans (MS1) were performed in the Orbitrap (AGC target 1e6, 120,000 resolution, 100 ms maximum injection time) and used to select the 10 most abundant features for MS/MS (MS2) analysis. Candidate peaks were filtered based on charge state ≥ 2 and mono-isotopic peak assignment, and dynamic exclusion (60 s ± 10 p.p.m.) was enabled. For non-phosphorylated peptide analysis, only one charge state was selected for each precursor. Precursor ions were isolated (AGC target = 2.5 × 10^4^) at a width of 0.5 Th using a quadrupole mass filter and fragmented with collision-induced dissociation (35 normalized collison energy (NCE)) in the ion trap with distinct maximum injection time settings for non-phosphorylated (150 ms) and phosphorylated (200 ms) peptides. To alleviate the effects of precursor ion interference^23^, multiple fragment ions were isolated^24^ using synchronous precursor selection (SPS) before higher-energy C-trap dissociation HCD (55 NCE, SPS notches = 8, AGC target = 2.2 × 10^5^, maximum injection time of 150 ms or 300 ms for non-phosphorylated and phosphorylated peptides, respectively) MS3 fragmentation and Orbitrap analysis (50,000 resolution).

A compilation of in-house software was used to convert Thermo “.raw” mass spectrometric data to mzXML format, as well as to correct mono-isotopic m/z measurements and erroneous peptide charge-state assignments^41^. The SEQUEST algorithm was used to assign MS/MS spectra to a peptide identification^42^. Static modifications included TMT (229.16293 Da) on both the N terminus of peptides and lysine residues, and carbamidomethylation of cysteine residues (57.02146 Da). Phosphorylation (79.96633 Da) was included for phosphopeptide experiments. Peptide spectral matches were filtered to 1% false discovery rate (FDR) using the target-decoy strategy^43^, before being grouped into proteins which were then filtered to 1% FDR at the protein level^41^. Phosphorylation sites were localized with a modified version of the AScore algorithm and phosphorylation sites with an AScore > 13 (*p* < 0.05) were considered localized^44^. Proteins and phosphorylation isoforms were quantified according to ref. ^40^. “Relative abundance” expression values for each analyte (protein or phosphorylation isoform) and represent the signal-to-noise value of each sample divided by the sum of all samples for each analyte normalized to 100. For phosphorylated peptides the quantitative values were normalized to the relative abundance of the protein, to account for changes in protein abundance upon treatment. All data analysis was performed using R (http://www.R-project.org). GO enrichment for protein and phosphoprotein data was performed using DAVID^45^.

### Bioinformatic analyses

Processing of microarray data: Affymetrix MoGene-2_0-st microarray data were processed using R packages and BioConductor. The raw hybridization signals were normalized using the robust multichip average method^46^ and transformed to log2 values. The SVA method^47^ was then used to remove undersigned variations.

Analysis of long vs. short isoforms of PDGF-A: The RNA sequencing gene expression datasets of all TCGA cancer types were downloaded from the Broad GDAC Firehose (stddata__2015_06_01, https://gdac.broadinstitute.org/). The expression levels of PDGF-A isoforms in 35 TCGA tumor types were analyzed. The percentage expression of each isoform was calculated and shown together with the expression of PDGFRα and PDGFRβ. In addition, PDGF-A expression in 53 tissue types were downloaded from the GTEx portal (http://www.gtexportal.org) and analyzed in the same way. Gene expression analysis of tumors compared with human tissue and cancer data: Geneset enrichment of the Verhaak et al.^3^ subtype signatures was calculated on our mouse models, as well as TCGA GBM patients.

Expression of ligand and receptor in our data relative to human tumors: To determine whether the expressions of PDGF-A and PDGFRα in our mouse model were localized in a physiologically relevant range compared to human patients, we compared the gene expression levels across mouse and human. To achieve an unbiased comparison, we performed rank normalization on mRNA expression profiles of both mouse samples and TCGA patient samples. Then the normalized expressions of PDGF-A and PDGFRα were compared with those in TCGA patient data to obtain a relative ranking.

### Live-cell imaging

For time-lapse experiments, cells were plated onto No 1.0 glass coverslip bottom multi-well plates (MatTek), and then placed in low serum media (0.1% heat-inactivated FBS in DMEM). After 24 h, 0, 0.1, or 10 μg mL^−1^ DOX was added to the cells. After 48 h of DOX treatment, vehicle or 100 nM VB was added to the cells and cells were imaged for 48 h. Live-cell imaging was conducted using an InCELL Analyzer 6000 (GE Healthcare Life Sciences) equipped with heated stage, heated lid, and constant CO_2_ flow. Twelve images per well/condition were acquired every 20 min for 48 h. These were performed in biological duplicates with at least nine independent FOVs counted in each replicate. For experiments investigating nuclear envelope breakdown, cells were plated and treated as above, with the addition of 50 nM of SiR-Tubulin (Cytoskeleton, Inc) 4 h before the addition of vehicle or VB and the start of imaging. Twelve images per well/condition were then acquired every 10 min for 8 h. Image analysis and quantification was done using Image J Image Processing Software.

### Cell viability and growth analysis

For viability analyses of murine GBM primary cultures, cells were placed into low serum media (0.1% heat-inactivated FBS in DMEM), and then 24 h later 0, 0.1, or 10 μg mL^−1^ DOX was added. Forty-eight hours after DOX treatment, vehicle or drug was added (Vinblastine Sulfate, Calbiochem; Vincristine Sulfate, Sigma Aldrich; Paclitaxel, Sigma Aldrich; Docetaxel, Sigma Aldrich; Ponatinib, Axitinib, Gefitinib, Crenolatinib, Telatinib, Amuvatinib, and Imatinib LC Laboratories). At the specified time points following the addition of drug (24, 48, 72, or 96 h), cells and supernatant were collected, and live and dead cells were counted using trypan blue exclusion. Survival is reflected as the percentage of live cells relative to the number of total cells counted. Viability assays were conducted in biological triplicates. For human PDX cultures survival assays, GBM6 cells^33^ were grown as monolayer, placed in low serum media (0.1% heat-inactivated FBS in DMEM) for 24 h and stimulated with 25 ng mL^−1^ PDGF-AA (Sigma Alrich) replenished every 12 h for 48 h. For the human GBM tumor sphere PDX cultures, cells (10^6^ cells/T75 non-adherent flask (Thermo Scientific)) were seeded in defined glioma stem cell medium (Neurobasal media (Invitrogen) supplemented with 20 ng mL^−1^ EGF (PeproTech), 20 ng mL^−1^ basic fibroblast growth factor (PeproTech), 2% B27 (Invitrogen)) and treated with increasing concentrations of VB for 48 h. Live cells were counted using trypan blue exclusion. Viability assays were conducted in biological triplicates.

### Mouse xenograft studies

For GBM6 xenograft studies, the cells were modified to express human PDGF-A ligand in an autocrine/paracrine manner by infection with a lentivirus expressing hPDGF-A in a DOX-dependent manner as described above. Cells were selected and tested in vitro for DOX-inducible PDGFRα kinase activity and reduction of phospho-STMN1 (Ser16). Single-cell suspensions were prepared in 1:1 volume 10^6^ cells:matrigel and injected subcutaneously in the flanks of athymic nude (Ncr^Nu/Nu^) mice (6–8weeks). Tumor size was measured with electronic calipers every 3 days and mice weights were determined at the same time. Tumor volume was calculated as follows: length × width^2^ × 0.5. Xenograft tumors formed within 4–5 weeks and were allowed to grow to ~300 mm^3^. When tumor volume reached approximately 100 mm^3^, mice were randomized and enrolled in the indicated treatment regimens (DOX diet of 625 mg kg^−1^ and/or VB (0.5 mg kg^−1^ IP every 3 days). Tumor numbers and volumes at the time of treatment initiation were: − DOX/− VB (*N* = 5) 96.8 ± 29.8 mm^3^, − DOX/ + VB (*N* = 5) 81.2 ± 40.1 mm^3^, + DOX/-VB (*N* = 5) 66.0 ± 24.3 mm^3^, + DOX/ + VB (*N* = 5) 98.7 ± 34.8 mm^3^. For treatment of mouse PDGFRα GBM-bearing mice with VB, 10^5^ PDGFRα mouse GBM cells, STMN1 CRISPR/Cas9 knockout derivative line, and modified GBM6 PDX were stereotactically injected intracranially in athymic nude (Ncr^Nu/Nu^) mice (6–8weeks) and put on a diet of DOX (625 mg kg^−1^), and their weights recorded daily. Upon loss of 8–10%, body weight (determined as tumor detection) treatments with VB (0.5 mg kg^−1^ IP every 3 days) were initiated. Animals were monitored to establish Kaplan–Meier survival curves. Experiments were conducted in two separate cohorts of mice.

### CRISPR/Cas9 knockout of STMN1

Six sgRNA sequences were derived from mouse STMN1 mRNA sequence as described in Supplementary Methods. sgRNAs were cloned in a version of pX330^48^ modified with a P2A-puromycin selection cDNA in frame with the Cas9 coding sequence. Plasmids were transfected into cells using Mirus Biotech reagent according to manufacturer’s protocol. Cells were selected with puromycin for 24 h and 7 days later individual clones were expanded and tested for STMN1 protein expression by western blotting.

### Cell cycle analysis

Cell cycle analysis was conducted in biological triplicate. Cells were placed in low serum media (0.1% Heat-inactivated FBS in DMEM) and then 24 h later 0, 0.1, or 10 μg mL^−1^ DOX was added. Forty-eight hours after DOX treatment, vehicle or 100 nM VB was added. Eight hours after drug treatment, 1 million cells were pelleted, washed in PBS, fixed in cold ethanol for 20 min, rehydrated in PBS, and then stained with propidium iodide nucleic acid stain for 15 min at room temperature (Invitrogen) per manufacturer’s protocol. Directly after staining, DNA content was analyzed using a Beckman Coulter Galieos with a 22 mW Argon 488 nM Laser. The percentage of cells in G1, S, and M phase were calculated using FlowJo Flow Cytometry Analysis Software.

### Statistical analyses

Statistical analyses were carried out using GraphPad Prism 7. Two-tailed Student’s *t*-tests were used for single comparison. Significance for survival analyses was determined by the log-rank (Mantel–Cox) test. *P*-values of < 0.05 were considered statistically significant.

### Data availability

Data that support the findings of this study have been deposited in Gene Expression Omnibus with accession GSE114438. All other relevant data are available from the corresponding author upon request.

## Electronic supplementary material

Supplementary Information

Description of Additional Supplementary Files

Supplementary Movie 1
