# Supplementary material for: A PDGFRα-driven mouse model of glioblastoma reveals a stathmin1-mediated mechanism of sensitivity to vinblastine
Source: Nat Commun. 2018 Aug 6;9:3116. doi: 10.1038/s41467-018-05036-4 (PMC6078993; doi:10.1038/s41467-018-05036-4)
Supplement: Supplementary file 1 — Supplementary Information [file 41467_2018_5036_MOESM1_ESM.pdf]

SUPPLEMENTARY INFORMATION

SUPPLEMENTARY METHODS

Conditional PDGFRα Transgenic Strain

The strategy for the construction of the PDGFRα-based genetically engineered mouse strains is as follows: wild-type human PDGFRα cDNA (a kind gift of Dr. Andrius Kazlauskas, The Schepens Eye Research Institute, Department of Ophthalmology, Harvard Medical School, Boston, Massachusetts.) was inserted in the CAGGS-Col1α1 vector plasmid (a kind gift from R. Jaenisch, Whitehead Institute, Cambridge, MA) using routine molecular biology techniques. After DNA sequencing for integrity, the CAGGS-PDGFRα-Col1α1 plasmid and a pCAGGS-Flpe plasmid were co-electroporated into C2 ES cells (R. Jaenisch, Whitehead Institute, Cambridge, MA). Clonal selection was achieved using hygromycin, and individual clones were screened by Southern blot hybridization with probes described elsewhere [1] (Supplementary Figure 1a). ES clones with properly knocked-in PDGFRα transgene (Supplementary Figure 1b) were used to produce chimeric mice, which were then mated to generate founder animals. Germline-transmitted LSL-hPDGFRα founder males were mated to conditional *Tp53* mice [2]. Compound LSL-hPDGFRα;p53$^{lox/lox}$ transgenic mice that were not exposed to Cre recombinase displayed no abnormalities. The LSL-hPDGFRα transgenic mice were genotyped by PCR using genomic DNA isolated from tail biopsies using the following primer set: Col frt A1 (5'GCA CAG CAT TGC GGA CAT GC3'), Col frt B (5'CCC TCC ATG TGT GAC CAA GG3'), and Col frt C (5'GCA GAA GCG CGG CCG TCT GG3') for the collagen1α1 locus genotype. The PCR cycling parameters are 94°C 5 min, 35 cycles at 94°C for 30 sec, 55°C for 30 sec, and 72°C for 30 sec followed by a 10-min extension at 72°C. Genotyping protocol for the Tp53 conditional strain was carried out as described elsewhere [2].

Virus Construct Design, Production and Titer Determination

We modified the pSLIK (single lentivector for inducible knock-down) vector system [3] to express the human PDGF-A cDNA and Cre recombinase. Our modificiation of the original lentiviral vector platform supports constitutive expression of a Tet-transactivating component (rtTa3) with the cDNA for Cre recombinase for in vivo experiments (Fig. 1) or blasticidine resistance gene for in vitro uses. Viruses are produced by cotransfection of 293T cells with packaging vectors and purified by ultracentrifugation of conditioned media, resuspended in PBS, aliquoted in single use amounts and stored at -80°C. To standardize intracranial injections with identical viral titers, viral preparations are functionaly titered for Cre activity by serial dilution infection of immortalized ear fibroblasts derived from Cdkn2a-null conditional LSL-tdTomato mice (Ai9 reporter strain) [4].

Intracranial Stereotactic Injections

Adult animals (3 months of age and older) of the indicated genotype were anesthetized with an IP injection of ketamine/xylazine (ketamine 100-125 mg/kg, xylazine 10–12.5 mg/kg). The animals were mounted in a Stoelting stereotaxic frame (Harvard Apparatus Inc.) with nonpuncturing ear bars. The incision site was shaved and sterilized with betadine surgical scrub, and a single incision was made from the anterior pole of the skull to the posterior ridge. A 1-mm burr hole was drilled at the stereotactically defined location of the striatum (2.1 mm rostral to the bregma, 1.5 mm lateral to the midline, and at 2 mm depth to the pia surface) and either a 1µl Hamilton syringe or a pulled glass pipette mounted onto a Nanoject II injector (Drummond Scientific Company) was used to inject the lenti-PDGFA-Cre virus at a rate of 0.1 uL/min. Following retraction of the syringe or pipette, the burr hole was filled with sterile bone wax, the skin drawn up and sutured, and the animal placed in a cage with a padded bottom atop a surgical heat pad until ambulatory.

Cell Viability

For survival in H1703 cells, cells were placed into low serum media (0.1% Heat Inactivated FBS in DMEM), and then 24 hours later stimulated with 25 ng/mL PDGF-AA (Sigma Alrich) every 12 hours for 48 hours. Cells were then treated with either vehicle or 100nM vinblastine and counted 24 hours later for live and dead cells using trypan blue exclusion. Survival is reflected as the percentage of live cells relative to the number of total cells counted. Viability assays were conducted in biological triplicates.

CRISPR/Cas9-Mediated Knock Out of STMN1

The sequence of the sgRNA are as follows:

		sequence
exon3	Guide #1	GAGCTGGAGAAGCGCGCTTC
	Guide #2	GGAGAAGCGCGCTTCAGGCC
exon4	Guide #1	TGGCCGCCATCTGCGCCTCC
	Guide #2	GGAAGTCTTGAAGCAGCTCG
exon5	Guide #1	CAGCCTCAGTCTCATCCGCG
	Guide #2	AGTCTCATCCGCGGGGTCTT

Gene Expression Analysis via qRTPCR

Cells pellets were isolated in biological triplicates, and total RNA was then isolated using the RNeasy Kit from Qiagen. Following RNA isolation, cDNA was then generated using Superscript III First Strand Synthesis Supermix (Thermo Fisher). Gene Expression was then analyzed using SYBR Green Real Time PCR with the following primers: 5'-TGCTGCTCCTCGGCTGCGGATA-3' and 5'-AAATGACCGTCCTGGTCTTGC-3' for the detection of Human PDGF-A and 5'-TTGGGGAGAGTGAAGTGAGCT-3' and 5'-GATGTAAATGTGCCTGCCTTC-3' for the detection of Human PDGFRα.

Histology and Immunohistochemistry

Deeply anesthetized animals were transcardially perfused with cold PBS. Brains were excised, rinsed in PBS, and serial coronal sections cut using a brain mold. Half of the sections were used to isolate primary cultures of tumor cells as described previously, and the other half were postfixed in 4% paraformaldehyde overnight. Formalin-fixed tissues were embedded in paraffin, sectioned at 5–10 μM, and stained with H&E (Sigma) for histopathological analysis. For immunohistochemistry (IHC), cut sections were deparaffinized and rehydrated through xylenes and graded alcohol series and rinsed for 5 min under tap water. Antigen target retrieval solution (Dako, S1699) was used to unmask the antigen (microwave for 10 min at low power then cooled down for 30 min) followed by 3 washes with PBS for 5 min each. Quenching of endogenous peroxidase activity was performed by incubating the sections for 10 min in 0.3% H_2O_2 in methanol followed by PBS washes. Slides were preincubated in blocking solution [5% (vol/vol) goat serum (Sigma) in PBS 0.3% (vol/vol) Triton-X100] for 1 hr at room temperature, followed by mouse-on-mouse blocking reagent (Vector Labs, Inc.; MKB-2213) incubation for 1 hr. Primary antibody was incubated for 24 hr at 4C. Secondary antibodies used were biotinylated anti-rabbit or anti- mouse (Vector Labs, Inc.; 1:500) for IHC and were incubated for one hour at room temperature. All antibodies were diluted in blocking solution. All immunobinding of primary antibodies was detected by biotin-conjugated secondary antibodies and Vectastain ABC kit (Vector Labs, Inc.) using DAB (Vector Labs, Inc.) as a substrate for peroxidase and counterstained with hematoxylin. The following primary antibodies were used: PDGFRα (Cell Signaling, 5241, 1:1000), PDGFRα (Cell Signaling, 3174, 1:1000), p-PDGFRα (Tyr 754, Thermo Fisher 441008G,

1:1000), Ki67 (Novocastra, NCL-ki67, 1:1000), PDGF-A (Santa Cruz Biotechnology, sc-9974, 1:500), Olig2 (EMD Millipore, AB9610, 1:10000), Iba1 (Wako, 019-19741, 1:2000), GFAP (Dako, Z-0334, 1:1000), NeuN (Chemicon, MAB377, 1:1000).

Immunoblots

Western blots were performed as follows: cell lysates were prepared in biological triplicates using radioimmunoprecipitation (RIPA) buffer supplemented with 5 mM Na_3VO_4 (freshly made) and CompleteTM protease inhibitor mixture (Roche). Fifty micrograms of total cell lysates were separated by SDS-PAGE and electrotransfered to polyvinylidene fluoride (PVDF) membrane (Immobilon P; Millipore). Blots were blocked in Odyssey® Blocking Buffer (TBS, LiCOR) for 1 hr at room temperature on a shaker. Primary antibodies were added to blocking solution with 0.2% (vol/vol) Tween-20 and incubated overnight at 4 °C on a shaker. Blots were washed several times with TBS-T, and secondary antibodies (IRDye® 800CW Goat anti-Rabbit and IRDye® 680RD Goat anti-Mouse, LiCOR) were added at 1:10,000 dilution into blocking solution with 0.2% (vol/vol) Tween-20 and 0.01% (wt/v) SDS and incubated for 1 hr at room temperature on a shaker in the dark. Blots were then analyzed using an Odyssey LiCOR Imaging System, and expression levels were quantified using Image Studio Lite Image Analysis Software. The following primary antibodies used in these studies were obtained from Cell Signaling Technology: p-PDGFRα (Tyr 849)/p-PDGFRβ (Tyr 857; 3170; 1:1,000 dilution), p-PDGFRα (Tyr 762; 12022; 1;1,000 dilution), p-PDGFRα (Tyr 1018; 4547; 1:1,000 dilution), PDGFRα (3174, 1;1,000 dilution), PDGFRα (5241; 1:1,000 dilution), pSTMN1 (ser16; 3353; 1:500 dilution), Cleaved Caspase 3 (Asp 175; 9661; 1:1,000 dilution), Caspase 3 (9662, 1:1,000 dilution), p-EGFR (Tyr-1173; 4407; 1:1,000 dilution), EGFR (2232; 1:1,000 dilution), β-Actin (3700; 1:2,000 dilution), anti- β-tubulin (DM1A; T9026-Sigma; 1: 2,000 dilution), and Stathmin 1 (Abcam, ab52630; 1:500 dilution), anti-Stathmin 1 (phospho S25; 1:1,000 dilution) antibody (SAB4300180) Sigma-Aldrich, (phospho S38) antibody (AP0221; 1:500 dilution) ABclonal.

Immunofluorescence

Cells were cultured on chamber slides, placed into low serum media (0.1% Heat Inactivated FBS in DMEM) for 24 hours, and then 0, 0.1 or 10 μg/mL Doxycycline was added. After 48 hours, cells were treated with either vehicle or 100nM vinblastine. 24 hours later, cells were washed twice in PBS, fixed in cold methanol for 10 minutes at -20C, washed twice quickly in cold acetone, washed twice in PBS, and incubated with FITC conjugated Anti-α-Tubulin antibody (Thermo Fisher F2168) diluted 1:100 in PBS plus 3% BSA overnight at 4C. Cells were then washed in PBS and mounted with DAPI Slowfade Gold Antifade mounting media (Thermo Fisher). Images were acquired using a Zeiss LSM 880 Confocal Laser Scanning Microscope, and were analyzed and quantified using Image J Image Processing Software. At least 5 biological replicates were analyzed for each condition.

Analysis of co-occurrence

Co-occurance between PDGFRA amplification/overexpression and each mutation/deletion event that presenting in at least 5% of patients was examined by using Fisher's exact test. Overexpression cutoff of PDGFRA was determined by the 10% quantile of its expression in PDGFRA amplified patients. The gene expression, copy number variation, mutation call were obtained from the Broad GDAC Firehose (stddata__2015_06_01, https://gdac.broadinstitute.org/).

SUPPLEMENTARY MOVIE 1

Cells were treated with VB with and without hPDGFRα activation and imaged for 48 hours.

SUPPLEMENTARY REFERENCES

1. Beard C, Hochedlinger K, Plath K, Wutz A, Jaenisch R. Efficient method to generate single-copy transgenic mice by site-specific integration in embryonic stem cells. *Genesis* **44**, 23-28 (2006).

2. Marino S, Vooijs M, van Der Gulden H, Jonkers J, Berns A. Induction of medulloblastomas in p53-null mutant mice by somatic inactivation of Rb in the external granular layer cells of the cerebellum. *Genes Dev* **14**, 994-1004 (2000).

3. Shin KJ*, et al.* A single lentiviral vector platform for microRNA-based conditional RNA interference and coordinated transgene expression. *Proc Natl Acad Sci U S A* **103**, 13759-13764 (2006).

4. Madisen L*, et al.* A robust and high-throughput Cre reporting and characterization system for the whole mouse brain. *Nat Neurosci* **13**, 133-140 (2010).

5. McKinnon RD, Waldron S, Kiel ME. PDGF alpha-receptor signal strength controls an RTK rheostat that integrates phosphoinositol 3'-kinase and phospholipase Cgamma pathways during oligodendrocyte maturation. *J Neurosci* **25**, 3499-3508 (2005).

6. van Heyningen P, Calver AR, Richardson WD. Control of progenitor cell number by mitogen supply and demand. *Curr Biol* **11**, 232-241 (2001).

7. Woodruff RH, Fruttiger M, Richardson WD, Franklin RJ. Platelet-derived growth factor regulates oligodendrocyte progenitor numbers in adult CNS and their response following CNS demyelination. *Mol Cell Neurosci* **25**, 252-262 (2004).

8. Meijer DH*, et al.* Separated at birth? The functional and molecular divergence of OLIG1 and OLIG2. *Nat Rev Neurosci* **13**, 819-831 (2012).

9. Nishiyama A, Lin XH, Giese N, Heldin CH, Stallcup WB. Co-localization of NG2 proteoglycan and PDGF alpha-receptor on O2A progenitor cells in the developing rat brain. *J Neurosci Res* **43**, 299-314 (1996).

10. Cahoy JD*, et al.* A transcriptome database for astrocytes, neurons, and oligodendrocytes: a new resource for understanding brain development and function. *J Neurosci* **28**, 264-278 (2008).

11. Zhang Y*, et al.* An RNA-sequencing transcriptome and splicing database of glia, neurons, and vascular cells of the cerebral cortex. *J Neurosci* **34**, 11929-11947 (2014).

SUPPLEMENTARY TABLE 1

<table>
<tr><td colspan="2" rowspan="2">odds ratio = 3.089
p.value = 7.56e-05</td><td colspan="2">TP53 loss (mutation or deletion)</td></tr>
<tr><td>FALSE</td><td>TRUE</td></tr>
<tr><td rowspan="2">PDGFRA amplification + overexpression</td><td>FALSE</td><td>144</td><td>43</td></tr>
<tr><td>TRUE</td><td>41</td><td>38</td></tr>
</table>

Fisher’s exact test for co-occurance between PDGFRA amplification/overexpression and mutant TP53 from TCGA GBM data.

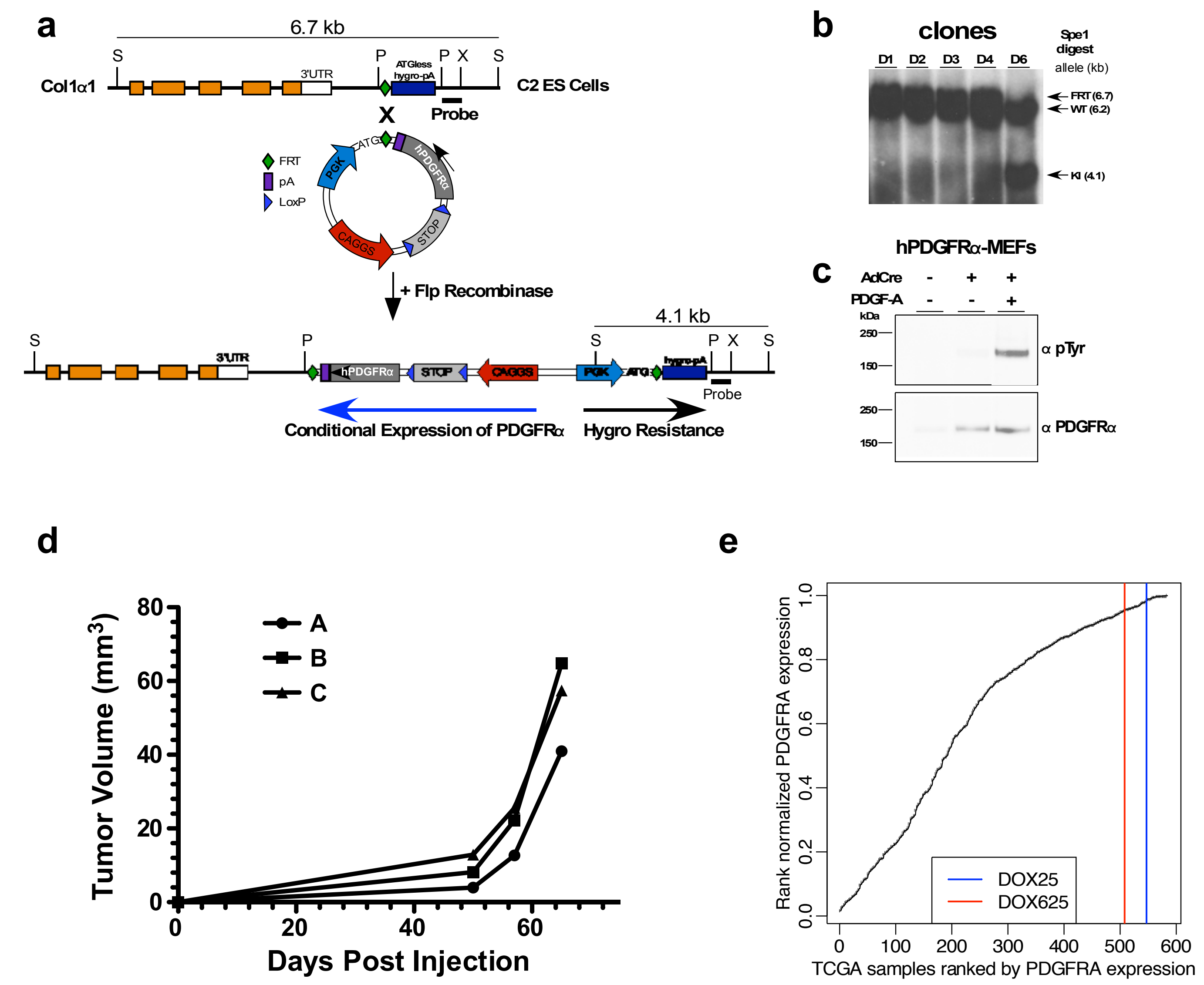

Supplementary Figure 1. Conditional expression of hPDGFRα.

(**a**) Schematic representation of the strategy to knock in the hPDGFRα cDNA into the 3'UTR of a modified collagen 1α1 locus (C2 mouse ES cells) using a Flp-in system (a kind gift of Drs. Marius Werning and Rudolph Jaenisch, Whitehead Institute). In these cells, a promoter and ATG-less hygromycin resistance cassette has been inserted in the 3' region of the collagen 1α1 gene. Transient co-transfection of a targeting plasmid and a Flpe recombinase plasmid in C2 ES cells result in the restoration of a functional hygromycin resistance cassette and the introduction of a CAGGS-loxSTOPlox-hPDGFRα segment. Col1α1 exons are shown as orange boxes and 3' UTR as an open box. P, PstI; S, SpeI; X, XhoI. Note that the figure is not drawn to scale. (**b**) Photomicrograph of a

southern blot analysis of hygromycin-resistant flp-in clones for hPDGFRα using a 3' internal probe (indicated in 1A). The size of DNA fragments (kb) representing the wild type (WT), FRT and knocked-in alleles are indicated. (**c**) Functional overexpression of hPDGFRα. Embryonic fibroblasts from a germline transmitted LSL-hPDGFRα knock in mouse were isolated and infected with adenovirus Cre to excise the LSL cassette and trigger the expression of hPDGFRα. Cells were treated with vehicle or recombinant human PDGF-A ligand at 50 ng/mL for 15 minutes. Cells were lysed and subjected to western blot analysis using anti-hPDGFRα and phospho-tyrosine antibodies as indicated. (**d**) Graphical representation of the quantification of tumor volumes from longitudinal MRI session over time demonstrates growth rates of three individual GBM tumors in vivo. (**e**) The achieved expression levels of hPDGFRα in our model are clinically relevant. Relative expression of hPDGFRα mRNA within our mouse GBM tumors is comparable to those overexpressed levels observed in human GBM.

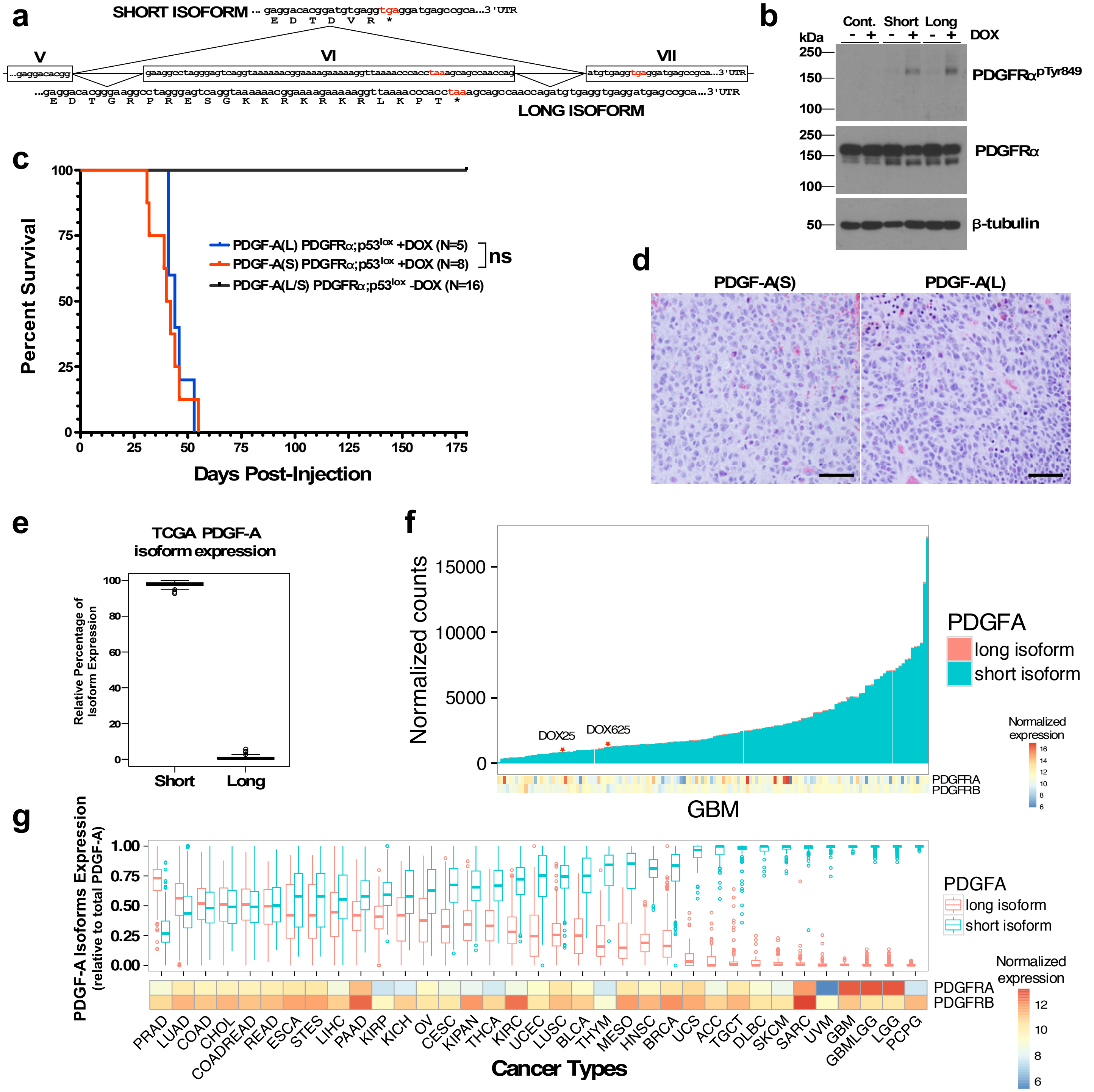

Supplementary Figure 2. Isoforms of hPDGF-A form tumors in mice.
(**a**) Schematic representation of exons V-VII of the human PDGF-A gene. The short (S) isoform mRNA arises from exon VI skipping giving rise to an exon V to VII in frame splice event which has a different C-terminal end from the long (L) isoform transcript, which retains exon VI. Exon VI codes for a basic amino acid stretch consisting of lysine and arginine residues. Introns-exons are not drawn to

scale. (**b**) Western blot analysis of Rat1 cells expressing hPDGFRα and either control vector or doxycycline-inducible (S) or (L) isoforms of hPDGF-A. Cells were treated with 10 μg/mL of DOX for 24 hours and subjected to immunoblot analysis using the hPDGFRα autophosphorylation site pTyr849 as an indirect measure of kinase activity, and anti hPDGFRα and β-tubulin as loading controls. Both forms are equally capable of stimulating the receptor's tyrosine kinase activity in cells in a DOX-inducible manner. (**c**) Absolute dependence on hPDGF-A expression for tumor formation. Tumor-free survival (Kaplan-Meier) analysis of hPDGF-A(S)-Cre- or hPDGF-A(L)-Cre-injected conditional LSL-hPDGFRα;$p53^{2lox}$ or LSL-hPDGFRα cohorts of mice treated with the indicated DOX (control or 250 mg/kg) diet. The survival analysis demonstrates identical latency and penetrance in tumor formation in both (L) and (S) isoforms of hPDGF-A. It also demonstrates that simply overexpressing hPDGFRα is not oncogenic even when presented on a loss of p53 tumor suppressor gene function, ns: not significant. (**d**) hPDGF-A (S) and (L) isoforms-derived tumors are histologically identical. H&E stained FFPE sections of long and short hPDGF-A driven tumors. Scale bar=50 μm. (**e**) The short isoform of PDGF-A is predominantly expressed in normal human tissues and patient gliomas. The expression of the two isoforms of PDGF-A was obtained from TCGA GBM RNA-seq level3 data. Boxplot shows the percent distribution of different isoforms for the 169 patient samples with RNA-seq data available plotted as percentage of all PDGF-A isoforms reported. PDGF-A(S) 97.8%, PDGF-A(L) 0.7%. The remaining 1.5% (not shown) corresponds to a never-reported, ill-defined third PDGF-A isoform. (**f**) The achieved expression levels of hPDGF-A in our model are clinically relevant. PDGF-A (S) and (L) expression in human GBMs. For each GBMs, normalized expressions of PDGFRα and PDGFRβ are indicated. The levels of hPDGF-A mRNA from GBMs derived from low (25 mg/kg) and high (625 mg/kg) DOX treated hPDGF-A;hPDGFRα;$p53^{lox}$ mice obtained from microarray data were anchored to those of human GBM patients as described in Experimental Procedures. Low DOX treated mice express less hPDGF-A than high DOX treated mice and both levels are within the physiologically relevant range. (**g**) Relative expression levels of both (S) and (L) isoforms of PDGF-A in 35 cancers. ACC (Adrenocortical carcinoma), BLCA (Bladder urothelial carcinoma), BRCA (Breast invasive carcinoma), CESC (Cervical and endocervical cancers), CHOL (Cholangiocarcinoma), COAD (Colon adenocarcinoma), COADREAD (Colorectal adenocarcinoma), DLBC (Lymphoid Neoplasm Diffuse Large B-cell Lymphoma), ESCA (Esophageal carcinoma), GBM (Glioblastoma multiforme), GBMLGG (Glioma), HNSC (Head and Neck squamous cell carcinoma), KICH (Kidney Chromophobe), KIPAN (Pan-kidney cohort (KICH+KIRC+KIRP)), KIRC (Kidney renal clear cell carcinoma), KIRP (Kidney renal papillary cell carcinoma), LGG (Brain Lower Grade Glioma), LIHC (Liver hepatocellular carcinoma), LUAD (Lung adenocarcinoma), LUSC (Lung squamous cell carcinoma), MESO (Mesothelioma), OV (Ovarian serous cystadenocarcinoma), PAAD (Pancreatic adenocarcinoma), PCPG (Pheochromocytoma and Paraganglioma), PRAD (Prostate adenocarcinoma), READ (Rectum adenocarcinoma), SARC (Sarcoma), SKCM (Skin Cutaneous Melanoma), STES (Stomach and Esophageal carcinoma), TGCT (Testicular Germ Cell Tumors), THCA (Thyroid carcinoma), THYM (Thymoma), UCEC (Uterine Corpus Endometrial Carcinoma), UCS (Uterine Carcinosarcoma), UVM (Uveal Melanoma). For each cancers, normalized expressions of PDGFRα and PDGFRβ are indicated. Interestingly, the (S) isoform is predominantly expressed in cancers with high expression levels of PDGFRα but not PDGFRβ. For boxplots, the center line represents the median, the bound box the interquartile range, and the whiskers lower quantile -1.5x interquartile range and upper quantile +1.5x interquartile range. For scatter plots, the center line represents the mean and upper and lower lines S.D.

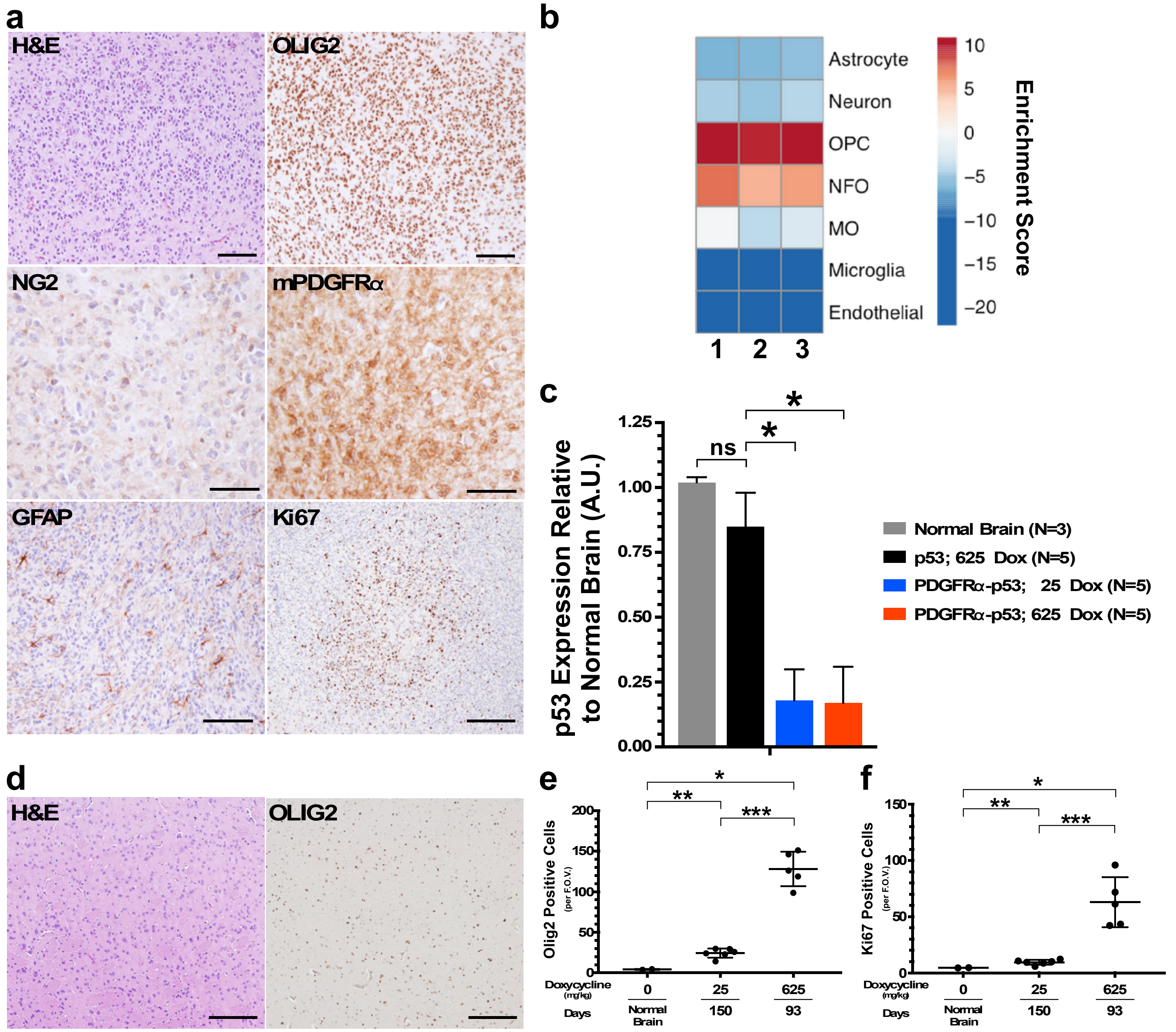

Supplementary Figure 3. PDGF-A expression in mice drives oligodendrocyte precursor cells (OPCs) proliferation. Ectopic CNS expression of PDGF-A in mice elicits a dose-dependent proliferation of PDGFRα-positive OPCs [5, 6, 7]. Consistent with this view, the hPDGF-A;$p53^{2lox}$ mice fed a high DOX diet displayed an oligodendrocytic histology and uniformly expressed **(a)** OPC markers PDGFRα, Olig2 [8] and NG2 [9] and were negative for GFAP. Representative photomicrographs of IHC from FFPE sections from hPDGF-A-Cre;$p53^{-/-}$ brains. Scale bar, 500 μm (H&E, OLIG2, Ki67), 250 μm (NG2, mPDGFRα), 200 μm (GFAP). **(b)** To substantiate the OPC nature of these cells, we profiled their transciptome of brains from hPDGF-A-Cre lentivirus injected $p53^{2lox}$ mice fed a high DOX diet and performed a gene set enrichment analysis (GSEA) against profiles obtained from primary isolates of mouse astrocytes, neurons, OPCs, resting oligodendrocytes, stimulated oligodendrocytes, microglia and endothelial cells [10, 11]. The highest enrichment scores were to those of pure OPCs and oligodendrocytes, reinforcing the notion that these are composed of proliferating OPCs responding to

exogenous hPDGF-A ligand. **(c)** The proliferating OPCs are not tumours. Cells from hPDGF-A-Cre lentivirus injected $p53^{2lox}$ mice fed a high DOX diet are incapable to grow ex vivo in culture conditions and they have a p53 wild type expression as revealed by qRT-PCR. Controls include primary cultures of P3 tumours, which are p53 null. (Error bars denote S.D. (n=3) *$p<0.0001$ Student's *t*-test). Lower expression of hPDGF-A (25 mg/kg DOX diet) resulted in fewer proliferating OPCs. DOX concentrations are in mg/kg in diet. **(d)** hPDGF-A-Cre lentivirus injected $p53^{2lox}$ mice fed a low DOX diet (25 mg/kg) do not display increased numbers of OPCs. Representative photomicrographs of H&E and Olig2 IHC from FFPE sections. Scale bar, 200 µm. **(e)** Percent Olig2 positive cells in (d), normal brain (n=2) and hPDGF-A-Cre lentivirus injected $p53^{lox}$ lesions from mice fed a low (25 mg/kg, n=6) and high DOX diet (625 mg/kg, n=5). (*$p<0.001$, **$p<0.004$, ***$p<0.0001$ by Student's *t*-test). **(f)** Ki67 IHC labeling proliferative index of normal brain (n=2) and hPDGF-A-Cre lentivirus injected p53lox lesions from mice fed a low (25 mg/kg, n=6) and high DOX diet (625 mg/kg, n=5). (*$p<0.02$, **$p<0.03$, ***$p<0.0003$ by Student's *t*-test). These results perhaps underscore the presence of a threshold level of PDGF-A below which OPC proliferation and recruitment is less vigorous. In fact, the sensitivity of OPCs to PDGF-A levels has been reported wherein differences in PI3K and pLCγ signaling pathways engagement were observed in low versus high PDGF in vitro [5]. Together, these results demonstrate that focal CNS expression of hPDGF-A support OPC accumulation through proliferation and recruitment of endogenous, p53 wild type OPCs in a dose-dependent manner, consistent with reported observations [6, 7]. For scatter plots, the center line represents the mean and upper and lower lines S.D.

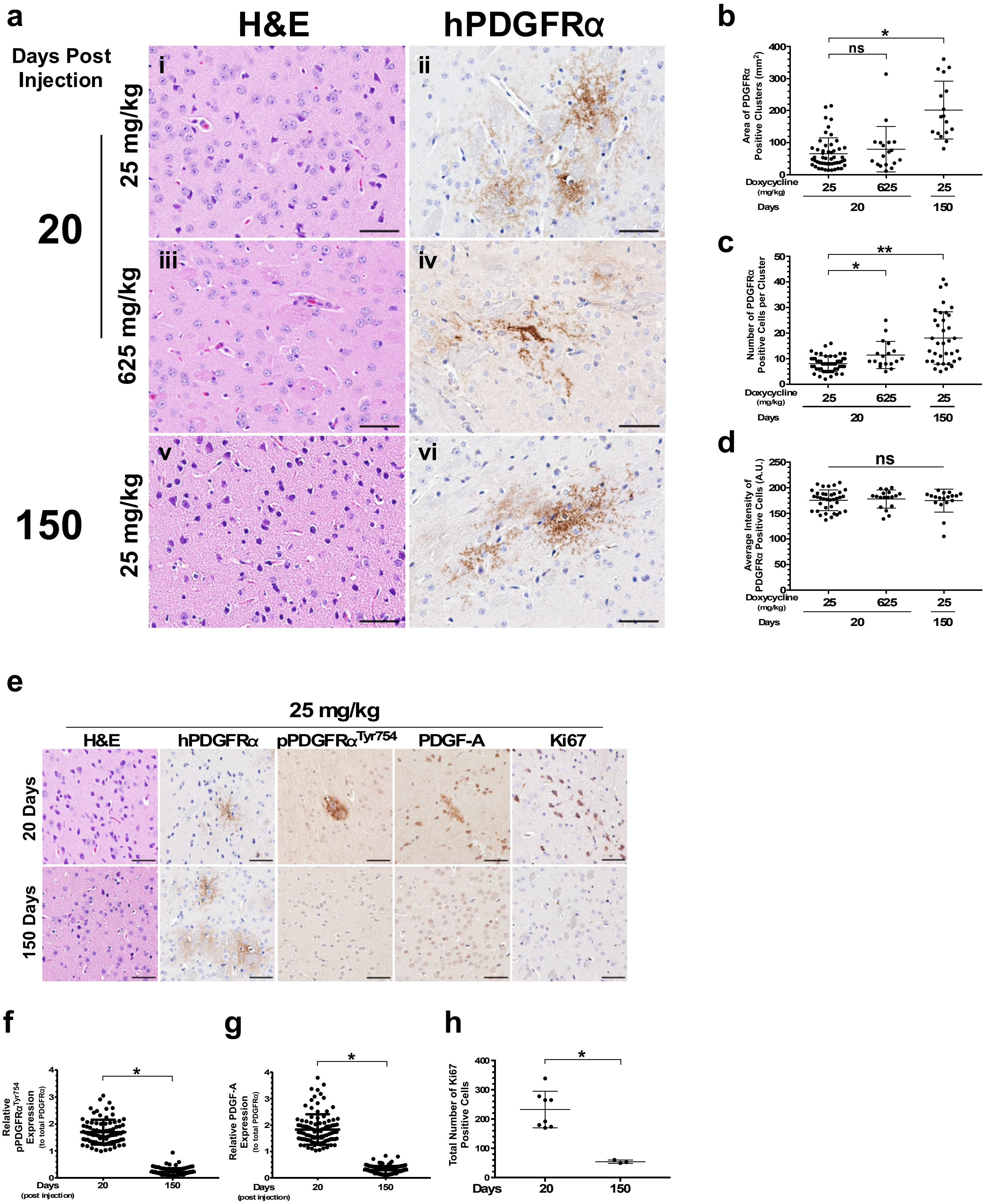
a
H&E
hPDGFRα
Days Post Injection
20
150
25 mg/kg
625 mg/kg
25 mg/kg
i
ii
iii
iv
v
vi
b
Area of PDGFRα Positive Clusters (mm2)
ns
*
Doxycycline (mg/kg)
Days
25
625
25
20
150
c
Number of PDGFRα Positive Cells per Cluster
*
**
d
Average Intensity of PDGFRα Positive Cells (A.U.)
ns
e
25 mg/kg
H&E
hPDGFRα
pPDGFRα Tyr754
PDGF-A
Ki67
20 Days
150 Days
f
Relative pPDGFRα Tyr754 Expression (to total PDGFRα)
Days (post injection)
g
Relative PDGF-A Expression (to total PDGFRα)
h
Total Number of Ki67 Positive Cells
Days

Supplementary Figure 4. Presence of indolent, residual hPDGFRα-positive clusters in low DOX-treated hPDGF-A;hPDGFRα;p53^{lox} mice.

(**a**) Presence of clusters of hPDGFRα-positive cells early post hPDGF-A-Cre lentiviral injection. Representative photomicrographs of FFPE processed brains of hPDGF-A-Cre lentivirus-injected hPDGFRα;p53^{2lox} mice 20 days post injection fed a low DOX diet (25 mg/kg) or a high DOX diet, and 150 days post injection fed a low DOX diet that did not succumb from GBM tumors stained with H&E or IHC using anti human PDGFRα antibody. Bar= 250 μm. (**b-d**) Graphical representations of quantifications of cluster attributes in (a). A minimum of 5 fields of view (F.O.V.) were used to quantitate (counts and intensity) PDGFRα positive cells and clusters. (**b**) Area (in mm^2) of the PDGFRα positive clusters, *p<0.0001 by Student's t-test. (**c**) number of PDGFRα positive cells per clusters, *p<0.002, **p<0.02 by Student's t-test and (**d**) expression levels of PDGFRα per cell, quantitated by densitometry, ns not significant by Student's t-test. We found that all the injected animals displayed similar numbers of PDGFRα-positive cells regardless of DOX concentration, and that these cells were found in small clusters of roughly the same sizes and numbers and with identical levels of hPDGFRα expression. These results demonstrate that identical Cre mediated recombination events occur in both DOX treatments, leading to similar hPDGFRα expression. (**e**) Representative photomicrographs of FFPE processed brains of hPDGF-A-Cre lentivirus-injected hPDGFRα;p53^{2lox} mice 20 days post injection and 150 days post injection that did not succumb from GBM tumors fed a low DOX diet (25 mg/kg) stained with H&E, or IHC using anti human PDGFRα, human PDGF-A, human phospho-PDGFRα Tyr 754 and the proliferation index marker Ki67 antibodies. Bar= 250 μm. (**f-h**) Graphical representations of quantifications of cluster attributes in (e). A minimum of 5 fields of view (F.O.V.) were used to quantitate (counts and intensity) phosphoPDGFRα, PDGFRα and ki67 positive cells and clusters. (**f**) expression of phospho-PDGFRα Tyr754 quantitated by densitometry relative to PDGFRα intensity, *p<0.0001 by Student's t-test (**g**) expression levels of PDGF-A quantitated by densitometry relative to PDGFRα intensity, *p<0.0001 by Student's t-test (**h**) proliferative index, total number of Ki67 positive cells. *p<0.001, Student's t-test. For scatter plots, the center line represents the mean and upper and lower lines S.D.

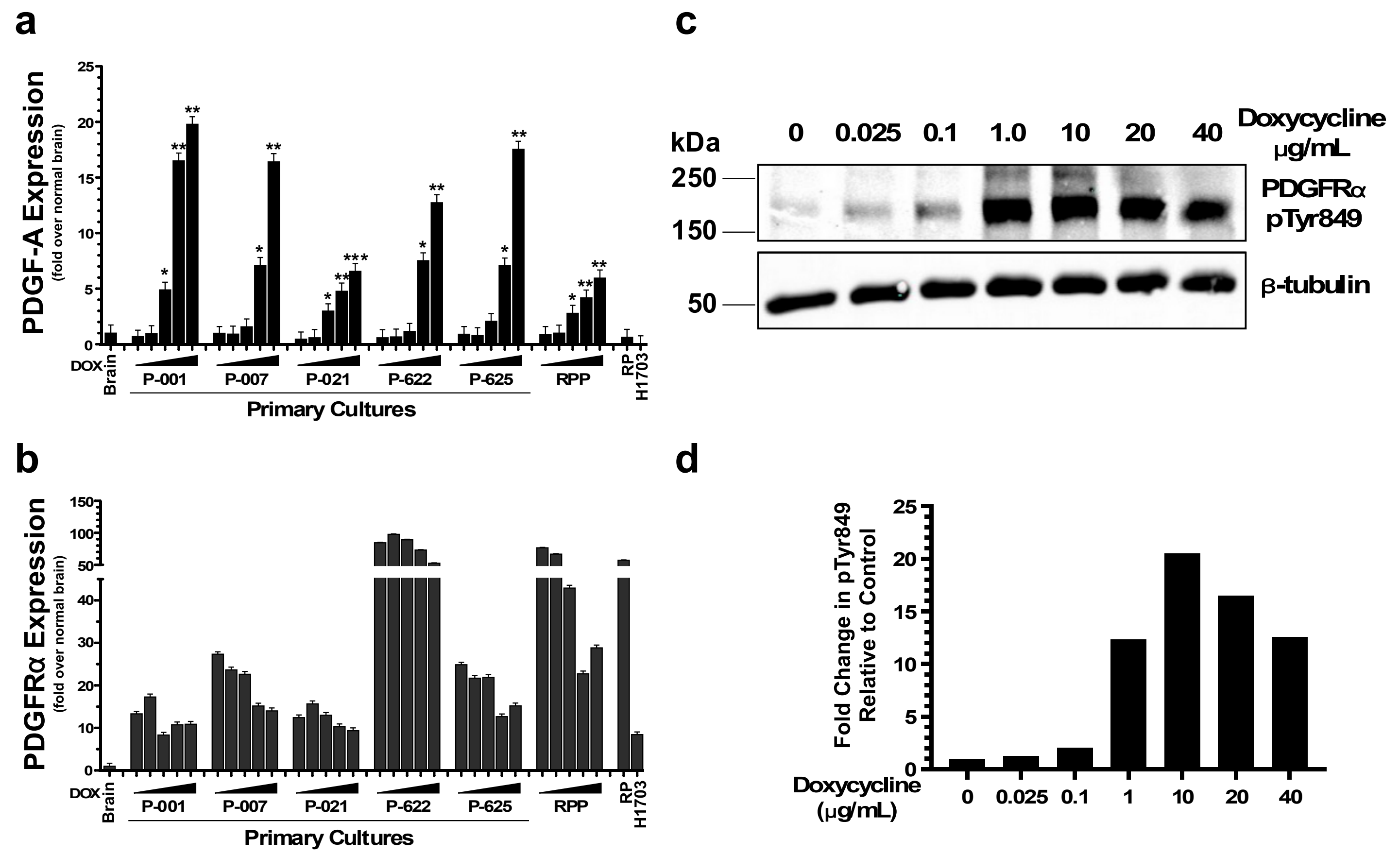

Supplementary Figure 5. hPDGF-A-Cre;hPDGFRα;p53^{lox} GBM primary cultures have titratable, DOX-inducible expression of hPDGF-A and activation of hPDGFRα.

Primary cell cultures from individual GBM tumors were established as described under Methods. Prior to incubation with different concentrations of DOX, cells were starved overnight in 0.1% FBS and treated with DOX for 48 hrs. RPP are Rat1 cells expressing hPDGFRα and infected with the DOX inducible hPDGF-A-Cre lentivirus. RP cells are Rat1 cells expressing hPDGFRα and H1703 is a NSCLC line that expresses endogenous levels of hPDGFRα. Cells were treated with 0, 0.01, 0.1, 1.0 and 10 μg/ml of DOX. The relative amount of ligand and receptors present in these primary cultures were measured by qRT-PCR quantitation of hPDGF-A (**a**) and hPDGFRα (**b**) mRNA. Data is average of biological triplicate, error bars S.D. Significance, P-001 *p<0.003, **p<0.0001, P-007 *p<0.0005, **p<0.0001, P-021 *p<0.03, **p<0.04, ***p<0.0007, P-622 *p<0.0004, **p<0.0001, P-625 *p<0.0005, **p<0.0001 and RPP *p<0.04, **p<0.006 ***p<0.002, all by Student's *t*-test. (**c**) Western blot analysis of P-021 cultures incubated in increasing concentrations of DOX for 48 hrs and probed for pTyr849 hPDGFRα, total hPDGFRα and control β-tubulin. (**d**) Graphical representation of the quantitation of band intensities in (c). We determined that concentrations of DOX (0.1 and 10.0 μg/mL) are capable to elicit lower and upper limits of detection of hPDGF-A mRNA levels and hPDGFRα activity (as measured by autophosphorylation on Tyr849).

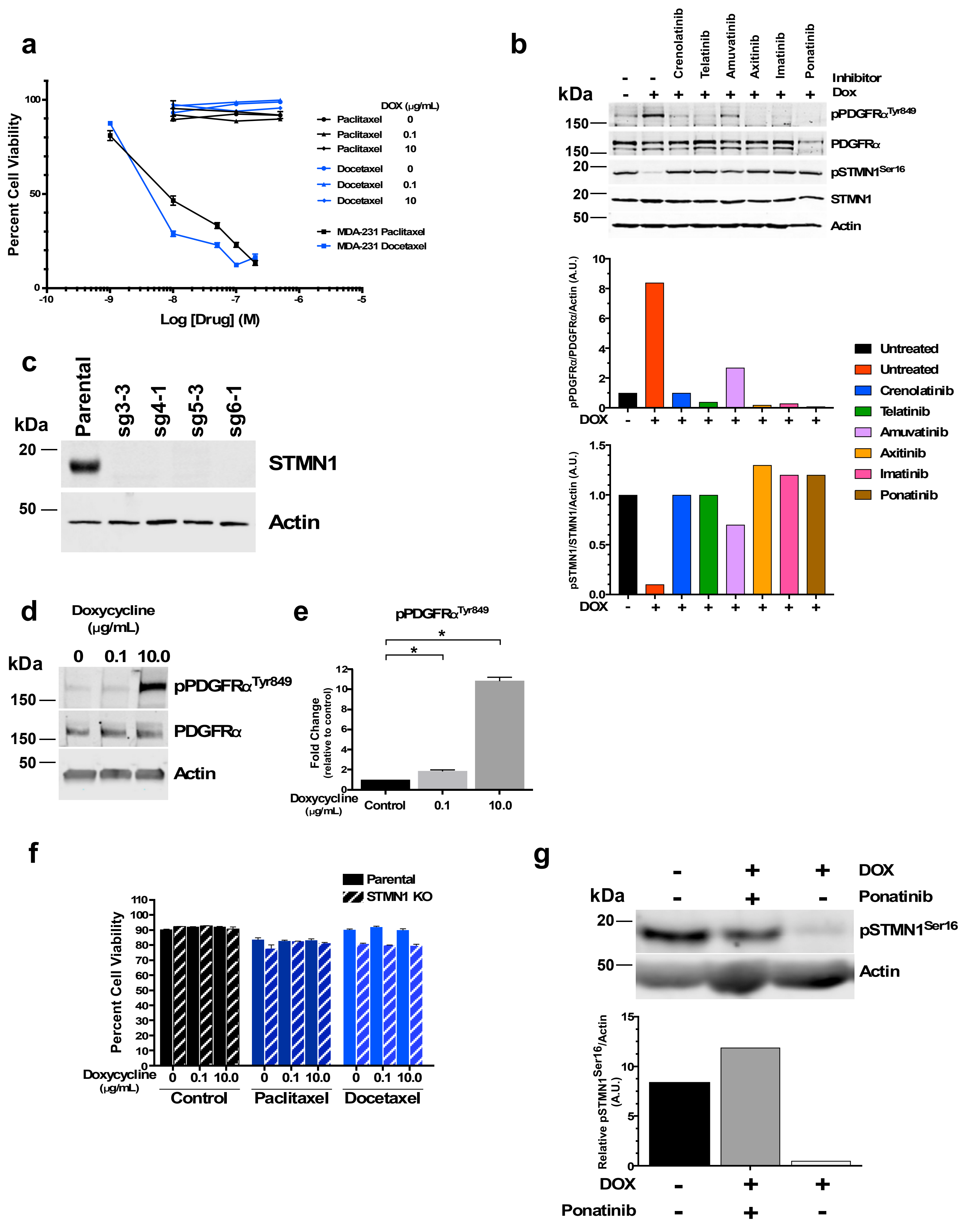
a
Percent Cell Viability
Log [Drug] (M)
DOX (μg/mL)
Paclitaxel 0
Paclitaxel 0.1
Paclitaxel 10
Docetaxel 0
Docetaxel 0.1
Docetaxel 10
MDA-231 Paclitaxel
MDA-231 Docetaxel
b
Crenolatinib
Telatinib
Amuvatinib
Axitinib
Imatinib
Ponatinib
Inhibitor
Dox
kDa
pPDGFRα Tyr849
PDGFRα
pSTMN1 Ser16
STMN1
Actin
pPDGFRα/PDGFRα/Actin (A.U.)
pSTMN1/STMN1/Actin (A.U.)
DOX
Untreated
Untreated
Crenolatinib
Telatinib
Amuvatinib
Axitinib
Imatinib
Ponatinib
c
kDa
Parental
sg3-3
sg4-1
sg5-3
sg6-1
STMN1
Actin
d
Doxycycline (μg/mL)
kDa
0
0.1
10.0
pPDGFRα Tyr849
PDGFRα
Actin
e
pPDGFRα Tyr849
Fold Change (relative to control)
Doxycycline (μg/mL)
Control
0.1
10.0
f
Parental
STMN1 KO
Percent Cell Viability
Doxycycline (μg/mL)
Control
Paclitaxel
Docetaxel
g
DOX
Ponatinib
kDa
pSTMN1 Ser16
Actin
Relative pSTMN1 Ser16/Actin (A.U.)

Supplementary Figure 6. PDGFR Inhibitors and the influence of STMN1 knock out on PDGFRα activation and sensitivity to taxanes.

(**a**) Dose response curve of cell viability to paclitaxel and docetaxel in MDA-231 and in absence and presence of hPDGFRα activity in hPDGFRα;p53$^{-/-}$ primary culture GBM cells. (**b**) Treatment with multiple inhibitors of PDGFR prevents phospho-STMN1 Ser16 reduction. Upper panel, western blot analysis of a PDGFRα positive mouse GBM culture treated with the indicated inhibitors (1 μM) for 24 hours and incubated with the indicated antibodies. Lower panel, graphical representation of quantitation of the western blot indicating ratio of phospho-PDGFRα Y849 over total PDGFRα normalized to actin levels relative to untreated samples and ratio of phospho-STMN1 Ser16 over total STMN1 normalized to actin and relative to untreated sample. (**c**) Western blot analysis of representative clones of PDGFRα positive GBM cultures knocked out for STMN1 using 4 individual sgRNAs. (**d**) Quantitative western blot of phospho-PDGFRα Tyr849 from a representative STMN1 CRISPR clone (sg3-3). (**e**) Graphical representation of quantitation of the western blot in (d) demonstrating retention of inducibility of PDGFRα kinase activity (as measured by Tyr849 autophosphorylation) by DOX in a STMN1 knock out clone. Data is average of biological triplicate, error bars S.D. n=3, *p<0.0001 by Student's *t*-test, when compared to no DOX control. (**f**) Elimination of STMN1 expression using CRISPR/Cas9 does not sensitize cells to taxanes regardless of hPDGFRα activity status. Cells (parental and STMN1 KO (clone sgRNA-4-1)) were incubated with 100 nM of paclitaxel or docetaxel for 96 hrs and cell viability determined by trypan blue exclusion assay. Data is average of biological triplicate, error bars S.D. (**g**) Inhibition of PDGFRα activity prevents reduction of phospho-STMN1 Ser16 levels. Western blot (upper panel) and quantitation (lower panel) of pSTMN1Ser16 relative to actin from PDGFRα;p53$^{-/-}$ GBM cells treated with DOX (10 μg/mL) and ponatinib (1μM) for 24 hours.

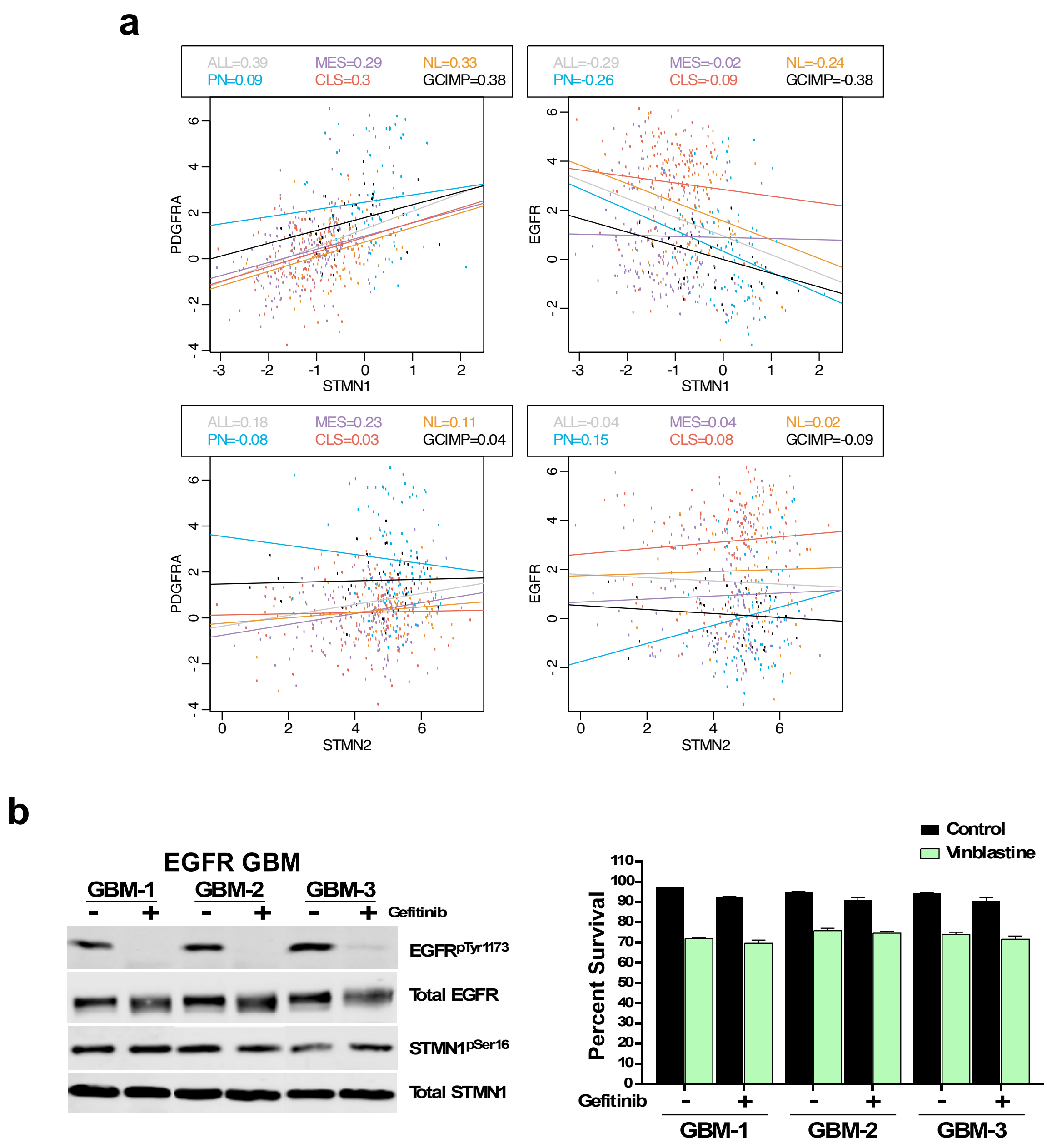

Supplementary Figure 7. PDGFRα but not EGFR mouse GBM tumors associate with STMN1 expression and sensitization to VB.

(**a**) Positive correlation of STMN1 and PDGFRα expression in GBM. Co-expression between PDGFRα/EGFR and STMN1/STMN2 in different subtypes of TCGA GBM patients. Pearson correlation coefficients are shown. All: all TCGA GBM patients; MES: Mesenchymal patients; NL: Neural patients; PN: Proneural patients; CLS: Classical patients; GCIMP: GCIMP patients. (**b**) STMN1 Ser16 phosphorylation is not controlled by EGFR in GBM, EGFR positive GBM cells are not sensitive to vinblastine. (error bars denote S.D. n=3).

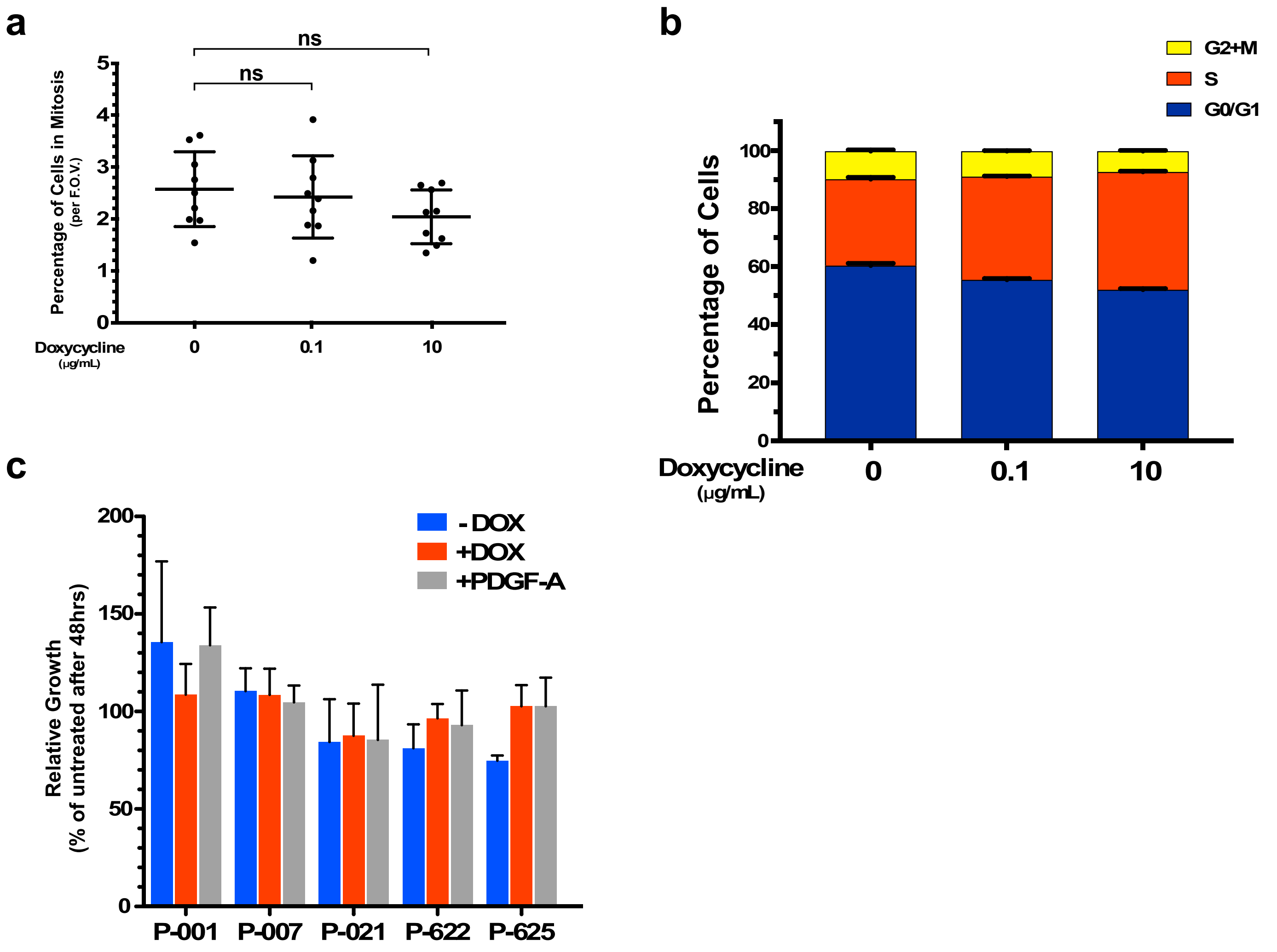

Supplementary Figure 8. hPDGF-A stimulation and mitosis.

(**a**) Chronic stimulation of hPDGFRα positive GBM cell culture with PDGF-A does not increase the percentage of cells in mitosis. For each treatment, n=9 field of view (FOV) were used to determine the percentage of cells in mitosis, ns, not significant by Student's *t*-test. (**b**) Cell cycle profile of a PDGFRα;p53$^{-/-}$ GBM primary culture treated with indicated amount of DOX for 24 hours. Data is average of biological triplicate, error bars S.D. (**c**) Cell growth assays of 5 PDGFRα;p53$^{-/-}$ GBM primary cultures treated with DOX (10 µg/mL) or exogenous PDGF-AA ligand (25 ng/mL) for 48 hours. Data is average of biological triplicate, error bars S.D. For scatter plots, the center line represents the mean and upper and lower lines S.D.

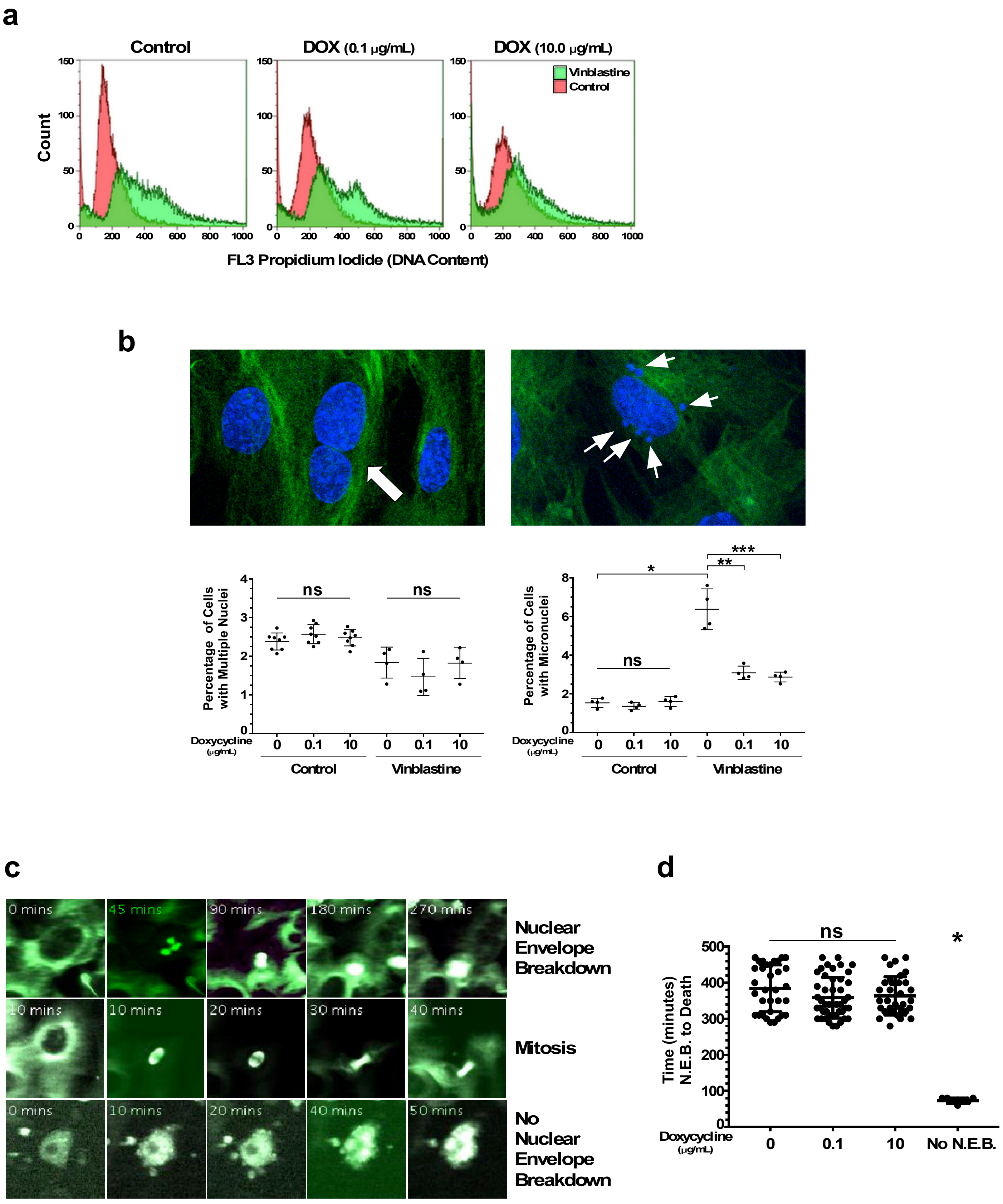
a
Control
DOX (0.1 μg/mL)
DOX (10.0 μg/mL)
Vinblastine
Control
Count
FL3 Propidium Iodide (DNA Content)
b
Percentage of Cells with Multiple Nuclei
ns
ns
Doxycycline (μg/mL)
Control
Vinblastine
Percentage of Cells with Micronuclei
*
**
***
ns
c
0 mins
45 mins
90 mins
180 mins
270 mins
Nuclear Envelope Breakdown
10 mins
10 mins
20 mins
30 mins
40 mins
Mitosis
0 mins
10 mins
20 mins
40 mins
50 mins
No Nuclear Envelope Breakdown
d
Time (minutes) N.E.B. to Death
ns
*
Doxycycline (μg/mL)
No N.E.B.

Supplementary Figure 9. Effects of hPDGF-A stimulation on gDNA content and percentage of micronucleated cells in PDGFRα positive GBM cultured cells.

(**a**) Representative flow cytometry plot assessing DNA content in vinblastine untreated and treated cells in control unstimulated, low hPDGF-A (0.1 μg/mL DOX) and high PDGF-A (10.0 μg/mL DOX) stimulation for 48 hours. (**b**) Representative photomicrographs of immunofluorescence images of PDGFRα positive GBM cultured cells treated with vinblastine and chronic PDGF-A stimulation depicting a multinucleated cell (Left panel, white thick arrow) and a cell with multiple micronuclei (right panel, thin white arrows). Lower panels, graphical representation of quantitation of the percentage of multinucleated (left panel) and micronucleated cells (right panel, *$p<0.0001$, **$p<0.001$, *$p<0.0006$). n=4 field of view were analyzed. ns; not significant, by Student's *t*-test. (**c**) Vinblastine and PDGFRα activity do not promote cell death outside of mitosis. Representative photomicrographs of time lapse microscopy of PDGFRα positive GBM cells incubated with SiR-tubulin (50 nM) binding fluorescent dye to visualize cell death independent of mitosis. Cells undergoing apoptosis without first entering mitosis have a breakdown of the nuclear envelop with fluorescently labeled tubulin entering the nucleus and a short time to cell death as opposed to SAC induced cell cycle arrest and metaphase stalling. (**d**) graphical representation of the quantitation of cells imaged in (d). ns; not significant, *$p<0.0001$ by Student's *t*-test. For scatter plots, the center line represents the mean and upper and lower lines S.D.

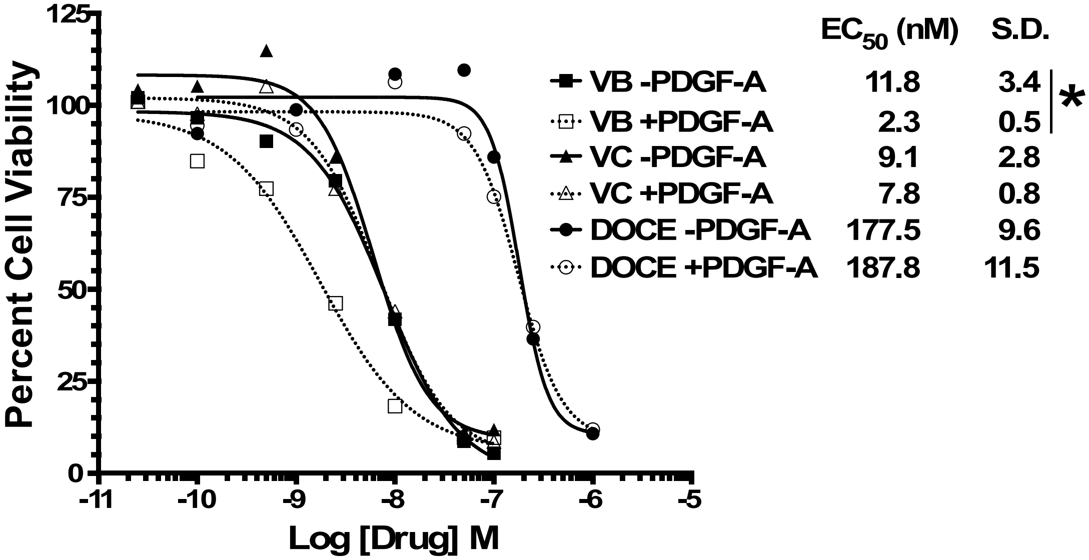

Supplementary Figure 10. Increased sensitivity to VB in GBM6 human GBM PDX upon PDGFRα activation.

Synthetic decreases in cell viability between PDGFRa activity and VB treatment but not vincristine or docetaxel. Dose response curves of VB, vincristine and docetaxel in the presence and absence of PDGF-A. Graph depicts representative curves of biological triplicates. S.D. n=3. *p<0.009 by Student's *t*-test.
