# Supplementary material for: A PDGFRα-driven mouse model of glioblastoma reveals a stathmin1-mediated mechanism of sensitivity to vinblastine
Source: Nat Commun. 2018 Aug 6;9:3116. doi: 10.1038/s41467-018-05036-4 (PMC6078993; doi:10.1038/s41467-018-05036-4)
Supplement: Supplementary file 2 — Description of Additional Supplementary Files [file 41467_2018_5036_MOESM2_ESM.pdf]

Description of Additional Supplementary Files

File Name: Supplementary Movie 1
Description: Cells were treated with VB with and without hPDGFRa activation and imaged for 48 hours.
